# Questions about NgAgo

**DOI:** 10.1007/s13238-016-0343-9

**Published:** 2016-11-15

**Authors:** Shawn Burgess, Linzhao Cheng, Feng Gu, Junjiu Huang, Zhiwei Huang, Shuo Lin, Jinsong Li, Wei Li, Wei Qin, Yujie Sun, Zhou Songyang, Wensheng Wei, Qiang Wu, Haoyi Wang, Xiaoqun Wang, Jing-Wei Xiong, Jianzhong Xi, Hui Yang, Bin Zhou, Bo Zhang

**Affiliations:** 1 National Human Genome Research Institute, NIH, Bethesda, MD 20892 USA; 2 Division of Hematology in Department of Medicine, Johns Hopkins University School of Medicine, Baltimore, MD 21205 USA; 3 Stem Cell Program in the Institute for Cell Engineering, Johns Hopkins University School of Medicine, Baltimore, MD 21205 USA; 4 Center for Vision Research, Eye Hospital, Wenzhou Medical University, Wenzhou, 325035 China; 5 Stem Cell and Functional Genomics Laboratory, School of Life Sciences, Sun Yat-sen University, Guangzhou, 510275 China; 6 HIT Center of Life Sciences, Harbin Institute of Technology, Harbin, 150080 China; 7 Department of Molecular, Cell and Developmental Biology, University of California Los Angeles, Los Angeles, CA 90095 USA; 8 Institute of Biochemistry and Cell Biology, Shanghai Institutes for Biological Sciences, Chinese Academy of Sciences, Shanghai, 200031 China; 9 State Key Laboratory of Stem cell and Reproductive Biology, Institute of Zoology, Chinese Academy of Sciences, Beijing, 100101 China; 10 College of Chemical Biology and Biotechnology, Peking University Shenzhen Graduate School, Shenzhen, 518055 China; 11 PKU BIOPIC, Beijing, 100871 China; 12 BIOPIC, ICG, CLS, and School of Life Sciences, Peking University, Beijing, 100871 China; 13 Center for Comparative Biomedicine, Institute of Systems Biomedicine, Shanghai Jiao Tong University, Shanghai, 200240 China; 14 Institute of Biophysics, Chinese Academy of Sciences, Beijing, 100101 China; 15 Institute of Molecular Medicine, Peking University, Beijing, 100871 China; 16 College of Engineering, Peking University, Beijing, 100871 China; 17 Laboratory of Disease Models in Non-Human Primates, Institute of Neuroscience, Shanghai Chinese Academy of Sciences, Shanghai, 200031 China; 18 College of Life Sciences, Peking University, Beijing, 100871 China

Dear Editor:

Gao et al. published data in Nature Biotechnology (Nat Biotechnol. 2016 May 2) showing that DNA-guided genome editing using the Natronobacterium gregoryi Argonaute (NgAgo) protein targeted 47 mammalian genomic loci with a 100% success rate and an efficiency of 21.3%–41.3% at various targets. This report led us to test NgAgo’s utility in various cells and organisms such as mouse and zebrafish for gene editing. In most cases, a codon-optimized NgAgo for vertebrate animals was first synthesized and tested with appropriate guide oligos targeting specific genes using techniques similar to what has been utilized for the CRISPR/Cas9 system. After failing to confirm any NgAgo induced genomic DNA editing in any experiments, some of us switched to use an NgAgo expression vector (CMV-NLS-NgAgo-SK) used and provided by Han, the senior author of this paper, available from Addgene (#78253) since June or directly from his lab. Again, no success editing endogenous genomic DNA was achieved. As controls, the ability of this construct to induce indels was tested, targeting the same genes in cultured human 293T cells as those reported in Fig. 4 of Gao et al. Several researchers in different laboratories independently performed the experiments but no indels were observed at targeted loci, as assayed by T7E1 digestion, PAGE and/or sequencing. Representative data that directly repeat Fig. 4 of Gao et al from eight laboratories are shown in Fig. 1 and protocols used are detailed in supplementary information. We also include additional results from testing NgAgo in various systems by laboratories of signees of this letter in supplementary information. None of these studies proves that NgAgo has any genome editing activities.

**Figure 1:**
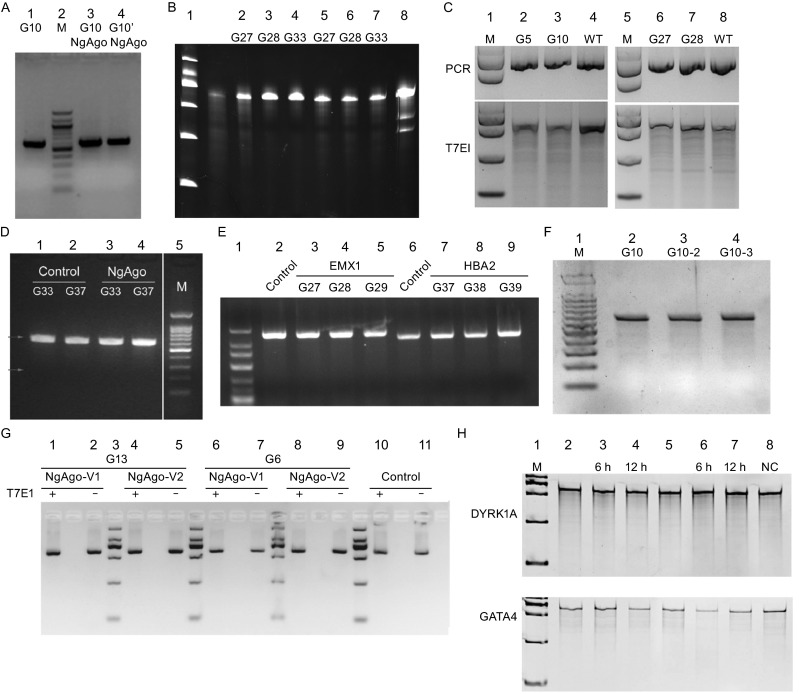
Results from repeating Fig 4 data of Gao et al using DNA guides with identical sequences and genomic targets. (A) T7E1 assay of NgAgo targeting DYRK1A using 293T cells. 1, Control, transfected with G10 only; 2, Marker; 3, Transfected with G10 and NgAgo; 4, Transfected with G10 plus G10 complementary oligo and NgAgo. (B) T7E1 assay of NgAgo targeting DYRK1A using 293T cells. 1, Marker; 2, 3 and 4, Controls transfected with G27, G28 or G33 guides only; 5, 6 and 7, Transfected with G27, G28 or G33 guides and NgAgo; 8, Positive control that confirms T7E1’s activity. (C) T7E1 assay of NgAgo targeting DYRK1A and EMX1 using 293T cells. 1 and 5, Marker; 2 and 3, transfected with G5 or G10 and NgAgo for DYRK1A; 6 and 7, transfected with G27 or G28 with NgAgo for EMX1. 4 and 8, Not transfected. Upper panel: PCR products only. Lower panel: T7E1 assay. (D) T7E1 assay of NgAgo targeting EMX1 and HBA2 using 293T cells. 1 and 2, transfected with G33 or G37 only; 3 and 4, transfected with G27 or G37 and NgAgo. (E) T7E1 assay of NgAgo targeting EMX1 and HBA2 using 293T cells. 1, Marker; 2 and 6, Control using a guide against GFP; 3, 4 and 5, transfected with G33 or G37 only; 3 and 4 transfected with G27, G28 or G29 and NgAgo for EMX1. 7, 8 and 9, transfected with G37, G38 or G39 and NgAgo for HBA2. (F) T7E1 assay of NgAgo targeting DYRK1A using 293T cells. 1, Marker; 2, Transfected with 500 ng G10 and 1 μg NgAgo; 3, Transfected with 1 μg G10 and 1 μg NgAgo; 4, Transfected with 500 ng G10 and 1 μg NgAgo, transfected 500 ng G10 again after 12 h. (G) T7E1 assay of NgAgo targeting DYRK1A using 293T cells. 1 and 6, Transfected with G13 or G6 and NgAgo-V1 for DYRK1A; 3, Marker; 4 and 8, Transfected with G13 or and NgAgo-V2 for DYRK1A; 2 and 7, Transfected with G13 or G6 and NgAgo-V1 for DYRK1A without T7E1; 5 and 9, Transfected with G13 or G6 and NgAgo-V1 for DYRK1A without T7E1; 10, Not transfected; 11, Not transfected without T7E1. NgAgo-V1: NLS-NgAgo-NLS. NgAgo-V2: NLS-NgAgo (codon optimization). (H) Surveyor assay of NgAgo targeting DYRK1A and GATA4 using 293T cells. 200 ng archaea codon NgAgo (aNgAgo) or codon humanized NgAgo (hNgAgo)-expressing plasmids were co-transfected with 500 ng G10 of DYRK1A or G41 of GATA4 gDNA into 293T cells respectively. gDNAs of DYRK1A and GATA4 were re-transfected 6 h or 12 h later as labeled. 1. Marker; 2, 3 and 4. aNgAgo; 5, 6 and 7. hNgAgo. 8. Not transfected control. Data sources: (A) Shuo Lin; (B) Zhiwei Huang; (C) Wei Li; (D) Jing-Wei Xiong; (E) Junjiu Huang and Zhou Songyang; (F) Wensheng Wei; (G) Hui Yang; (H) Haoyi Wang

Han issued public statements suggesting that the reported findings require “superb experimental skills” and one needs to be able to repeat the result of Fig. 3C, which is the inhibition of GFP expression in plasmid DNA transfected cells. Indeed, plasmid GFP expression reduction by co-transfection of NgAgo and its targeting DNA oligo is reproducible in our hands. However, we cannot demonstrate by sequencing this reduction is a result of DNA mutation. Many factors can affect this type of GFP expression, including NgAgo’s ability to target RNA as well as non-specific stress induced by oligo and DNA transfection. More recently, Han added that the activity of NgAgo is very sensitive to mycoplasma or bacteria in the culture. However, it seems unlikely that independent laboratories would all have their cells contaminated, resulting in consistently negative results for DNA editing activity. In fact, several of the signees of this letter have made sure that our cells are free of mycoplasma by first testing them before performing replication experiments.

The key point of paper by Gao et al is that DNA-guided NgAgo’s can efficiently target 47 genomic loci with a 100% success rate and a ≥20% efficiency. Neither the originally published protocol nor the newly released information on Addgene’s website involves any steps that seem to require “superb experimental skills”. To gain insights into NgAgo’s utility, some of us have even sent visiting researchers to Han’s laboratory but they were not allowed to perform genome editing experiments involving mammalian cells when they were there. Consequently, none of them returned with any information confirming Han’s data. Discussions on NgAgo have been frenzied in online forums, which cited some of the informal discussions in support of Han’s experimental data. Han also quoted David Cyranoski’s report (Nature, 2016 August 09) as evidence that NgAgo’s genome editing function had been confirmed. This further creates confusion because information in online forums is not accessible by the broader scientific community. We therefore urge the authors of the original paper to clarify the uncertainty surrounding NgAgo and provide all the necessary details for replicating the initial, very important results.

## Electronic supplementary material

Below is the link to the electronic supplementary material.

Supplementary material 1 (PDF 25975 kb)

