# Erratum to: Questions about NgAgo

**DOI:** 10.1007/s13238-016-0349-3

**Published:** 2016-12-02

**Authors:** Shawn Burgess, Linzhao Cheng, Feng Gu, Junjiu Huang, Zhiwei Huang, Shuo Lin, Jinsong Li, Wei Li, Wei Qin, Yujie Sun, Zhou Songyang, Wensheng Wei, Qiang Wu, Haoyi Wang, Xiaoqun Wang, Jing-Wei Xiong, Jianzhong Xi, Hui Yang, Bin Zhou, Bo Zhang

**Affiliations:** 1 National Human Genome Research Institute, NIH, Bethesda, MD USA; 2 Division of Hematology in Department of Medicine, Johns Hopkins University School of Medicine, Baltimore, MD 21205 USA; 3 Stem Cell Program in the Institute for Cell Engineering, Johns Hopkins University School of Medicine, Baltimore, MD 21205 USA; 4 Center for Vision Research, Eye Hospital, Wenzhou Medical University, Wenzhou, 325035 China; 5 Stem Cell and Functional Genomics Laboratory, School of Life Sciences, Sun Yat-sen University, Guangzhou, 510275 China; 6 HIT Center of Life Sciences, Harbin Institute of Technology, Harbin, 150080 China; 7 Department of Molecular, Cell and Developmental Biology, University of California Los Angeles, Los Angeles, CA 90095 USA; 8 Institute of Biochemistry and Cell Biology, Shanghai Institutes for Biological Sciences, Chinese Academy of Sciences, Shanghai, 200031 China; 9 State Key Laboratory of Stem cell and Reproductive Biology, Institute of Zoology, Chinese Academy of Sciences, Beijing, 100101 China; 10 College of Chemical Biology and Biotechnology, Peking University Shenzhen Graduate School, Shenzhen, 518055 China; 11 PKU BIOPIC, Beijing, 100871 China; 12 BIOPIC, ICG, CLS, and School of Life Sciences, Peking University, Beijing, 100871 China; 13 Center for Comparative Biomedicine, Institute of Systems Biomedicine, Shanghai Jiao Tong University, Shanghai, 200240 China; 14 Institute of Biophysics, Chinese Academy of Sciences, Beijing, 100101 China; 15 Institute of Molecular Medicine, Peking University, Beijing, 100871 China; 16 College of Engineering, Peking University, Beijing, 100871 China; 17 Laboratory of Disease Models in Non-Human Primates, Institute of Neuroscience, Shanghai Chinese Academy of Sciences, Shanghai, 200031 China; 18 College of Life Sciences, Peking University, Beijing, 100871 China

## Erratum To: Protein Cell DOI 10.1007/s13238-016-0343-9

In the original publication of this article the Figure 1E legend has been incorrectly published.

The authors would like to request the following replacement to the Figure 1E legend:

T7E1 assay of NgAgo targeting *EMX1* and *HBA2* using 293T cells. 1, Marker; 2 and 6, Transfection control using a GFP expression vector; 3, 4 and 5 transfected with G27, G28 or G29 and NgAgo for *EMX1*. 7, 8 and 9 transfected with G37, G38 or G39 and NgAgo for *HBA2*.

