# Supplementary material for: Questions about NgAgo
Source: Protein Cell. 2016 Nov 15;7(12):913–5. doi: 10.1007/s13238-016-0343-9 (PMC5205665; doi:10.1007/s13238-016-0343-9)
Supplement: Supplementary file 1 — Supplementary material 1 (PDF 25975 kb) [file 13238_2016_343_MOESM1_ESM.pdf]

Supplementary information

Protocols used for Fig panels in correspondence:

The Figure shows data from replication experiments of Fig 4 by Gao et al. We therefore have strictly followed the protocol from the original paper and the later released protocol on Addgene's website. Our specific attempts include using DNA oligos phosphorylated at 5' either by commercial vendors or in-house by individual laboratories, increasing transfection frequency and using cells free of mycoplasma. Since no success was achieved by both protocols, only representative figures are included.

Data from experiments involving other targets, cells, organisms and approaches:

We list data from several laboratories with their individual's information included.

NgAgo failed to cleave DNA targets in human 293T cells

Fei Teng[1], Bing Qu[1], Wei Li[1]

[1]State Key Laboratory of Stem cell and Reproductive Biology, Institute of Zoology, Chinese Academy of Sciences, Beijing 100101, China

Email: liwei@ioz.ac.cn

Summary of finding:

To detect whether NgAgo can site-specifically cleave DNA *in vitro*, we purified NgAgo protein, and previously reported TtAgo and MpAgo proteins from *Escherichia coli*, and performed single-strand DNA (ssDNA) cleavage supplied with guide DNAs and RNAs respectively. While the TtAgo and MpAgo site-specifically cleaved the DNA targets, the NgAgo failed to do so (**Figure 1A**). We also repeated Han's NgAgo-based precise genome mutagenesis experiments using the 293T cells, which were free of Mycoplasma infection examined before experiments. However, even using the same guide DNA sequences that can efficiently produce indels in Dyrk1a and Emx1 loci according to Han's paper, we failed to detect any targeted mutation by either T7E1 assay or sanger sequencing (**Figure 1B**). Hence we claim that we cannot repeat Han's results and NgAgo-based DNA site-directed mutagenesis does not work in our hands.

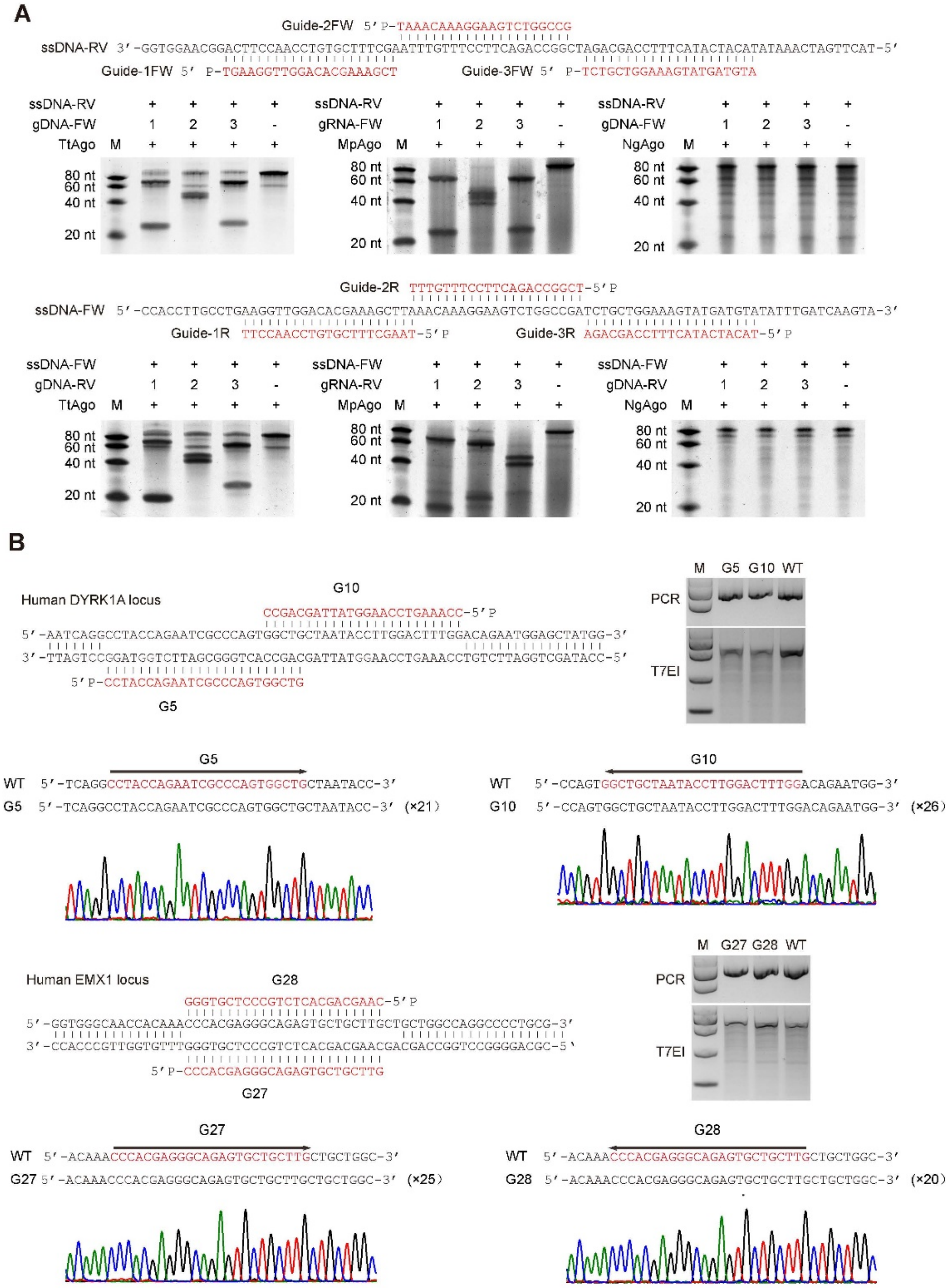

Figure legends

Figure 1. A) The 88-nucleotide (nt) ssDNA targets can be cleaved site-specifically by 5'-OH RNA guides and purified TtAgo and MpAgo protein at 75 °C and 50 °C, respectively. However, the same targets cannot be cleaved site-specifically by purified NgAgo protein and 5'PO_4-guide DNA at 37°C, 50 °C or 75 °C. **B)** NgAgo fails to produce indels in human 293T cells in Dyrk1a or Emx1 gene locus targeted by the same guide DNAs (G5, G10, G27, G28) from Han's paper.

Materials and Methods

Synthesis and purification of TtAgo, MpAgo and NgAgo. Codon-optimized TtAgo, MpAgo and NgAgo sequences were synthesis (Taihe Gene) and cloned into a custom pEASY-E1 expression vector (Transgen Biotech). N terminal 6x His-tagged Ago proteins were expressed in Transetta (DE3) chemically competent cell (Transgen Biotech). For protein purification, cells were grown in LB medium and to an OD600 of ~0.5, a final concentration of 1mM IPTG (isopropyl β-D-1-thiogalactopyranoside) were added for induction expression of 16h. Proteins were finally purification adapted to the manuscripts of Ni-NTA Spin Kit Handbook (Qiagen). Dialyzed proteins were used for for the following steps.

Oligonucleotide synthesis. Guide DNAs or RNAs of various lengths (19 nt to 25 nt) were synthesized. 5'-PO_4 modification of gDNA were added in the synthesis process (BGI) or using T4 polynucleotide kinase (NEB). 5'-OH or 5'PO_4 modification of gRNAs were directly synthesized (Ribobio).

In vitro cleavage assays. Purified Ago proteins, ssDNA targets, and DNA or RNA guides RNA guides were incubated in NEBuffer 2 (NEB). Various concentrations of MnCl2 or MgCl2 were supplied for activity assays. The reaction temperature of TtAgo and MpAgo was 75 ^{o}C and 50 ^{o}C, respectively. For NgAgo in vitro cleavage, the reported reaction buffer (10 mM Tris PH 8.0, 20 mM NaCl, 0.5 mM MgCl2, 0.4% glycerol, 2 mM DTT and 20 μg/ml BSA) from Han's paper was also tested with incubation temperature from 37 ^{o}C to 50 ^{o}C.

Genome editing assays. Different guide DNAs were co-transfected eukaryotic expression plasmid carrying NgAgo and GFP genes in 293T cells respectively. GFP positive cells were selected via flow cytometry sorting for genomic DNA extraction. The DNA fragment flanking the targeting sites were further amplified and adopted to T7EI assay. PCR products were also cloned into pEASY-T1 cloning vector and transformed into *E. coli* competent cells (Transgen Biotech) for Sanger sequencing.

NgAgo failed to inactivate GFP expression in HEK-293 cells

Haihua Xie[1], Feng Gu[1]

[1]School of Ophthalmology and Optometry, Eye Hospital, Wenzhou Medical University, State Key Laboratory Cultivation Base and Key Laboratory of Vision Science, Ministry of Health and Zhejiang Provincial Key Laboratory of Ophthalmology and Optometry, Wenzhou, Zhejiang 325027 China

Email address: fgu@mail.eye.ac.cn(F.G.).

Summary of finding:
We used EGFP reporter system to perform NgAgo-mediated genome editing in cultured human cells. No indels of *EGFP* DNA and reduction of GFP was detected.

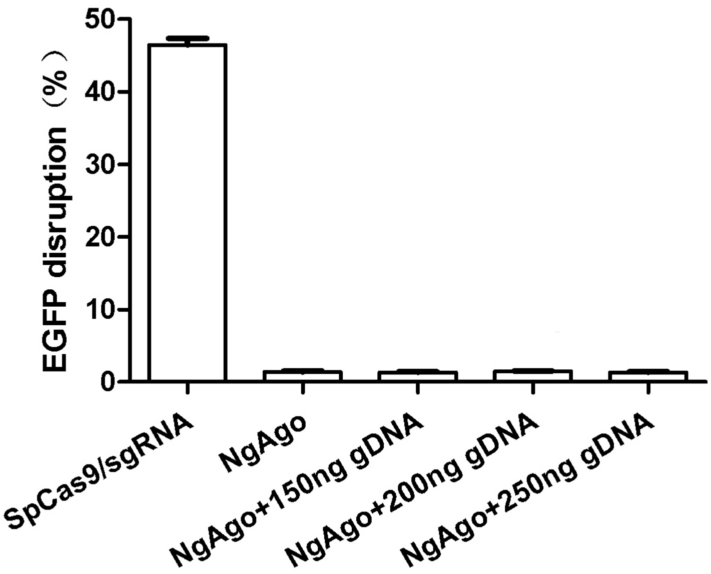

Figure 1. NgAgo failed to cleave *EGFP* in human cells

NgAgo expression plasmids with different amount of 5' phosphorylated ssDNA targeting *EGFP* gene were transfected into 293-SC1 cells, which carry a single copy of *EGFP* gene in the genome. The CRISPR/Cas9 (SpCas9) targeting the *EGFP* sequence partial overlapping with that of NgAgo was used as positive control. With different amount of

ssDNA(150ng, 200ng and 250ng) and NgAgo (500ng) expression plasmids of transfection, no functional inactivation of *EGFP* gene was detected (same as negative control). In contrast, the CRISPR/Cas9 group inactivated EGFP expression in more than 45% cells.

Methods

Generation and culture of 293-SC1 Cells

The lentiviral vector plasmid pSIN-*EGFP* containing an *EGFP* gene, IRES and Puromycin gene, was generated from pSIN-EF2-Lin28-Puro (obtained from Addgene; ID 16580) using EcoR I and BamH I restriction enzyme sites. HEK-293 cells were obtained from ATCC (CAT#CRL-1573), and grown at 37°C in 5% CO2 in Dulbecco's modified Eagle's medium (Life Technologies, Carlsbad, CA), 10% heat-inactivated fetal bovine serum, penicillin, and streptomycin.

HEK-293 cells expressing *EGFP* were generated by transduction with lentivirus at serial dilution and selection with puromycin (0.9 µg /ml) until all cells in control dishes had detached (6 to 8 days). Drug-resistant single colonies of transduced HEK-293 cells were isolated and confirmed to contain single copy of EGFP (named 293-SC1). To maintain *EGFP* expression, the medium for 293-SC1 culture included puromycin.

Transfection

We followed transfection procedure described by Gao et al. (Nat Biotechnol. 2016 May 2) to introduce NgAgo and CRISPR/Cas9 constructs and oligos. CRISPR/Cas9 plasmids were constructed as described online (http://www.genome-engineering.org/crispr/). The oligonucleotide sequences used are summarized in Tables S1. Plasmid DNA was isolated by standard techniques. NgAgo expression construct was generated by insertion of the coding sequence of NgAgo plus a NLS at N-terminus into mammalian gene expression vector as that used in Gao et al. (Nat Biotechnol. 2016 May 2). Constructs were all confirmed by sequencing.

Table S1. Target sequences.

Target sites for CRISPR/Cas9 or NgAgo System

Target site ID	Target sequence (5'-3')	PAM	Strand
NGG-1	CAAGTTCAGCGTGTCCGGCG	AGG	+
gDNA	**5'**-P-CAAGTTCAGCGTGTCCGGCGAGGG		+

NgAgo failed to cleave DNA targets in human cells, mouse embryonic stem cells and mouse embryos

Puping Liang, Zhou Songyang, Junjiu Huang

Stem Cell and Functional Genomics Laboratory and Key Laboratory of Gene Engineering of the Ministry of Education, School of Life Sciences, Sun Yat-sen University, China

Email address: songyanz@mail.sysu.edu.cn (Z.S.). and hjunjiu@mail.sysu.edu.cn (J.H.)

Summary of finding:
We performed experiments as described in Figure legend and Figure 1. No NgAgo mediated target DNA cleavage were detected in human cells, mouse embryonic stem cells and mouse embryos.

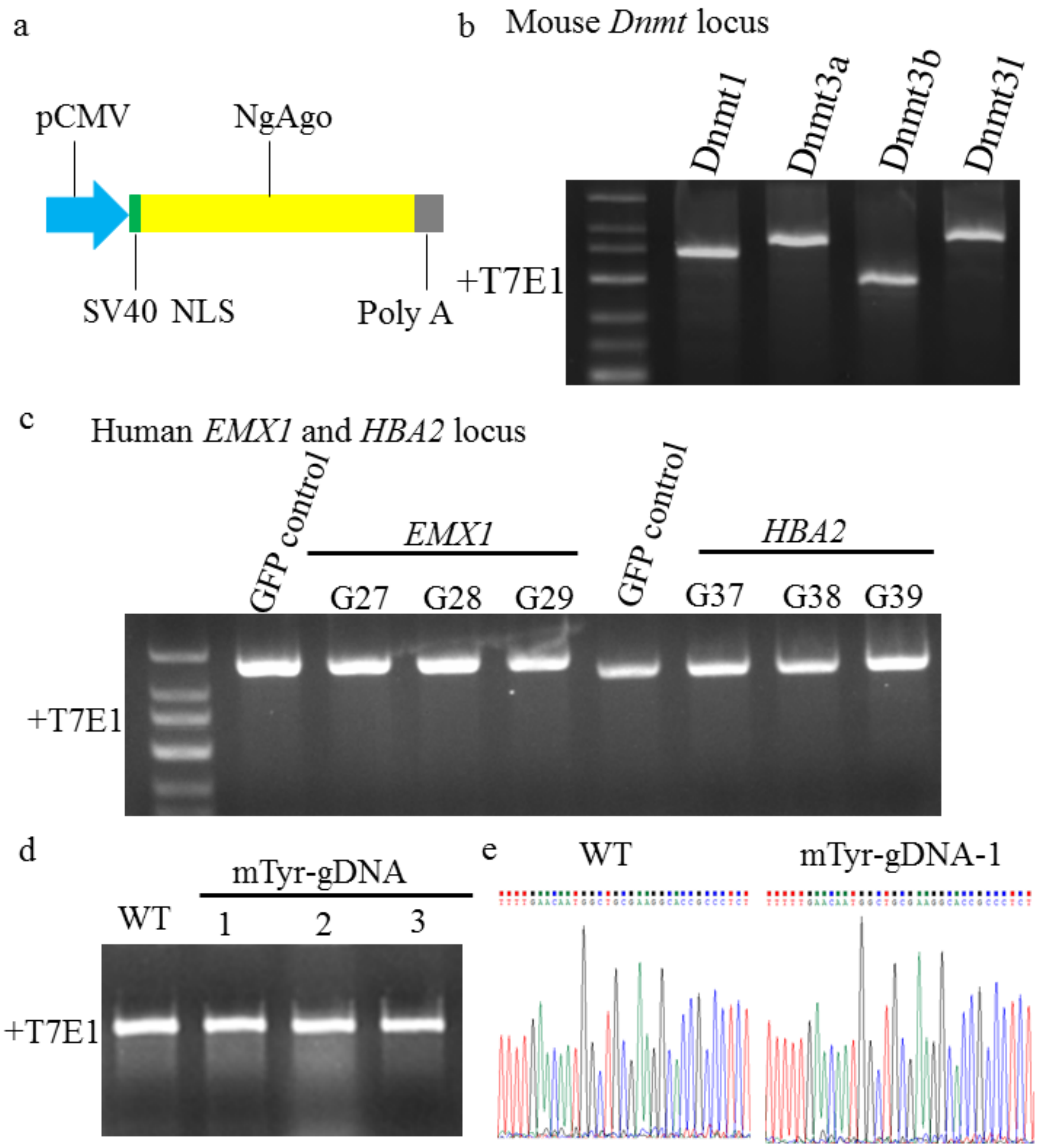

f

Human gDNA	Sequence
EMX1-G27	5' P-cccacgagggcagagtgctgcttg-3'
EMX1-G28	5' P-caagcagcactctgccctcgtggg-3'
EMX1-G29	5' P-gccaatggggaggacatcgatgtc-3'
HBA2-G37	5' P-gagatggcgccttcctctcagggc-3'
HBA2-G38	5' P-gcgccttcctctcagggcagagga-3'
HBA2-G39	5' P-ctcttctctgcacagctcctaagc-3'

Mouse gDNA	Sequence
mDnmt1	5' P-ccactaaacggaaacccaaggaag-3'
mDnmt3a	5' P-ggagacttggagaagcggagtgaa-3'
mDnmt3b	5' P-gacaggagatggagacagagatga-3'
mDnmt31	5' P-aggctgtgaccctccaggatgtcc-3'
mTyr-gDNA-1	5' P-tttgaacaatggctgcgaaggcacc-3'
mTyr-gDNA-2	5' P-ggccgataggtgcattggcttctgg-3'
mTyr-gDNA-3	5' P-gtctctgttatggccgataggtgc-3'

Figure 1. NgAgo could not cleave target gene in human cells, mouse embryonic stem cells (ESCs) and mouse embryos. (a) Schematic of the NgAgo expression vector used in human 293T cells and mouse ESCs. (b) T7E1 assay for NgAgo-mediated cleavage. 0.5 μg gDNA and 2 μg NgAgo Plasmid were transfected into mouse embryonic stem

cells by nucleofection. 48 hour later, cells were harvested and genomic DNA were extracted for T7E1 assay. Results showed that NgAgo could not cleave the *Dnmt* genes in mouse ESCs. (c) gDNA targeting *EMX1* and *HBA2* are from Han's paper. 0.5 μg gDNA and 2 μg NgAgo Plasmid were transfected into human 293T cells using Lipofectamine 3000. 48 hour later, cells were harvested and genomic DNA were extracted for T7E1 assay. Results showed that NgAgo could not cleave the targets in human cells. (d) 0.5-day zygotes were harvested from C57 mice. 100 ng/μl gDNA and 200 ng/μl NgAgo mRNA were injected into the cytoplasm. 10 injected embryos from each gDNA were cultured for 48 hours. 10 embryos were lysed as a mixture, and then *Tyr* locus were PCR amplified for T7E1 assay and Sanger sequencing. (e) Representative Sanger sequencing results of embryos resulting from *Tyr* NgAgo targeting. (f) Sequence of gDNA used in this study. All these gDNAs were ordered from Sangon Biotech (Shanghai) or Generay Biotech (Shanghai).

Negligible genome editing in human 293T cells by NgAgo-mediated guide DNA

Senquan Liu[1, 2], Linzhao Cheng[1, *]

[1]Stem Cell Program, Institute for Cell Engineering and Division of Hematology, Department of Medicine, The Johns Hopkins University School of Medicine

[2]The Zhijiang University School of Medicine

*Correspondence: lcheng2@jhmi.edu, Miller Research Building 747, 733 North Broadway, Baltimore, MD 21205, USA; Phone: 410-614-6958

Conflict-of-Interest Disclosure

The authors declare no competing interests

For more than a decade, the Cheng lab in JHU has been working on improving the efficiency of human genome editing using ZFNs, TALENs and CRISPR-Cas9 nucleases (1-7). Although nowadays the user-friendly CRISPR-Cas9 is very efficient in making mutations via non-homologous end joining (NHEJ) in human cancer cell lines such as 293T cells, the efficiency of achieving homologous recombination (HR) is still low (2-5%) in 293T cells and even lower in other biologically relevant cells such as human induced pluripotent stem cells (iPSCs) (6-7). A recent paper reported that the *Natronobacterium gregoryi* Argonaute (NgAgo) is able to mediate DNA-guided genome editing in human cell lines including 293T cells at a high efficiency (8). This led us to investigate if the NgAgo is able to achieve efficiently DNA-guided genome editing (HR) in human iPSCs as well as in 293T cells. We obtained an NgAgo expression vector (CMV-NLS-NgAgo-SK) used by Gao et al. (8) from Addgene (plasmid #78253). In addition, we obtained a similar expression vector that contains the full length NgAgo cDNA synthesized based on DNA sequences from the NCBI database (data not shown). However, we failed to detect any genome editing activities in any human cell lines tested, using the standard assays that we previously succeeded using ZFNs, TALENs and CRISPR-Cas9 (1-7).

The negative results prompted to conduct more experiments with positive controls and simple 293T cells that consistently show genome editing (HR) aided by the other three types of nucleases (1-7). Two previously validated CRISPR guide RNAs, T1 and T2, are used to targeting the AAVS1 locus, together with an expression vector expressing the spCas9 protein controlled by the same CMV promoter/enhancer as the one for NgAgo (9). For the DNA guides (D1 and D2), we synthesized single stranded DNA containing the same sequence recognized by T1 and T2, plus additional 4 nucleotides (nt) at the 3’ end to make the optimal 24-nt length as reported (8). The guide DNAs D1 and D2 were further phosphorylated at 5’ either by the vendor synthetically or by us using T4 polynucleotide kinase. Human 293T cells (free of mycoplasma, as confirmed by the MycoAlert kit purchased from Lonza) were used for detecting genome

editing of a common HR reporter system with different nucleases (**Fig 1A**). We have used this system previously for testing various forms of ZFNs, TALENs and CRISPR-Cas9, and HR efficiencies in 293T were consistently 2-5% (2, 4, 6-7).

We transfected the 293T cells that contain genomically integrated EG*IP-AAVS1 template with a promoter-less donor GFP DNA (2, 9), and with or without co-transfected spCas9/guide RNA vectors or the NgoAg vector and a guide DNA. Three days after, numbers of cells expressing GFP from the repaired GFP template was measured by flow cytometry (**Fig 1B**) as we did before (2, 9). The background level of false GFP+ cells is ~0.43% (Panel 1). The spCas9 together with either guide RNA T1 or T2 showed ~1.80% and 2.55%, respectively. However, co-transfection of the NgoAg expression vector or in combination with the guide DNA D1 or D2 (Panel 4-6) did not show significant activities over the background (**Fig 1B and 1C**). We observed similarly negative results (data not shown) of targeting human *HBB* sequence when we used NgAgo and guide DNAs, in comparison with that mediated by CRIPSR-Cas9, TALENs or ZFNs, targeting at the genomically integrated *HBB* reporter locus (2, 4, 5).

Although the AAVS1 reporter system we used for monitoring HR has limitations, it has an advantage avoiding pitfalls of DNA-based technology using PCR amplification of the targeted DNA sequence. The guide DNA of 24-nt remained in the cell lysate and after DNA isolation could serve as primer to generate a DNA fragment smaller than that using two flanking primers. By using this GFP reporter system, we avoided either the requirement of T7E1 or surveyor nuclease assay that demands an NHEJ efficiency of >5%, or false positive results due to errors by high-through put DNA sequencing and PCR amplification. Corroborating with many other data presented in this correspondence, we conclude that the genome editing (HR) activities conferred by the described NgoAg-mediated guide DNA system (8) is negligible even in the facile 293T human cell line.

Reference:

1. Zou J, Maeder ML, Mali P, et al. Gene targeting of a disease-related gene in human induced pluripotent stem and embryonic stem cells*.* Cell Stem Cell 2009, 5:97-110.

2. Zou J, Sweeney CL, Chou BK, et al. Oxidase deficient neutrophils from X-linked chronic granulomatous disease iPS cells: functional correction by zinc finger nuclease mediated safe harbor targeting. Blood, 2011; 117:5561-5572.

3. Zou, J., Mali, P., Huang, X., Dowey, S.N., and Cheng, L. (2011a). Site-specific gene correction of a point mutation in human iPS cells derived from an adult patient with sickle cell disease. Blood *118*, 4599-4608.

4. Yan, W., Smith, C., and Cheng, L. (2013). Expanded activity of dimer nucleases by combining ZFN and TALEN for genome editing. Sci Rep *3*, 2376.

5. Huang, X., Wang, Y., Yan, W., Smith, C., Ye, Z., Wang, J., Gao, Y., Mendelsohn, L., and Cheng, L. (2015). Production of Gene-Corrected Adult Beta Globin Protein in Human Erythrocytes Differentiated from Patient iPSCs After Genome Editing of the Sickle Point Mutation. Stem Cells *33*, 1470-1479.

6. Smith, C., Gore, A., Yan, W., Abalde-Atristain, L., Li, Z., He, C., Wang, Y., Brodsky, R.A., Zhang, K., Cheng, L.*, et al.* (2014). Whole-Genome Sequencing Analysis Reveals High Specificity of CRISPR/Cas9 and TALEN-Based Genome Editing in Human iPSCs. Cell Stem Cell *15*, 12-13.

7. Smith, C., Abalde-Atristain, L., He, C., Brodsky, B.R., Braunstein, E.M., Chaudhari, P., Jang, Y.Y., Cheng, L., and Ye, Z. (2015). Efficient and allele-specific genome editing of disease loci in human iPSCs. Mol Ther *23*, 570-577.

8. Gao F, Shen XZ, Jiang F, Wu Y, and Han C. DNA-guided genome editing using the Natronobacterium gregoryi Argonaute. Nature Biotech. 2016 Jul;34(7):768-73.

9. Mali, P., Yang, L., Esvelt, K.M., Aach, J., Guell, M., DiCarlo, J.E., Norville, J.E., and Church, G.M. (2013b). RNA-guided human genome engineering via Cas9. Science *339*, 823-826.

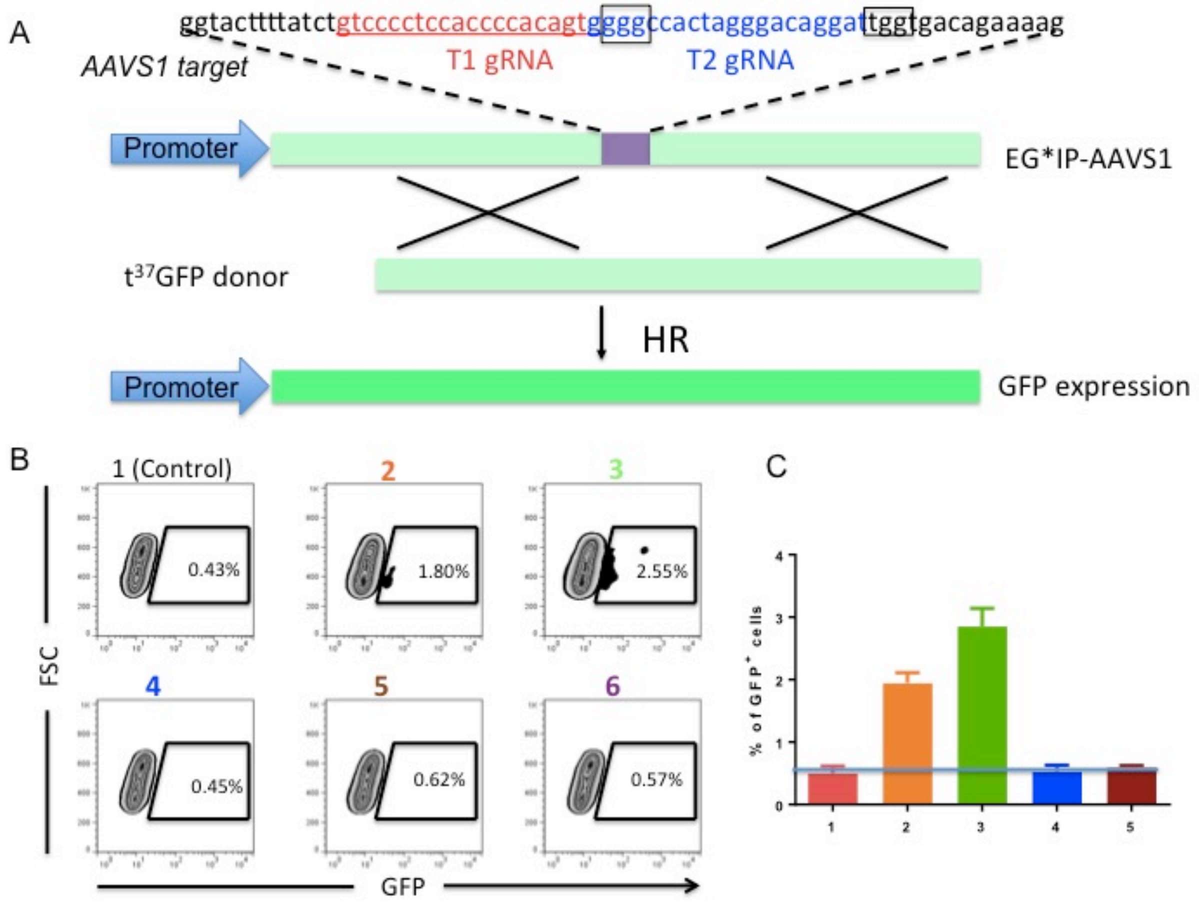

Figure 1. Negligible genome editing in human 293T cells by NgoAg-mediated guide DNA as compared to the CRISPR-Cas9 system.

A. Diagram of an HR reporter system testing various designed nucleases targeting the AAVS1 locus (2, 9). The two guide RNAs (gRNA) T1 (red) and T2 (blue) are denoted, together with their PAM sequences in boxes. The guide DNAs D1 and D2 contain the NGG PAM and an additional 1-nt at 3'. The genomically integrated reporter EG*IP-AAVS1 and a donor template t37-GFP were described before (9). **B**. Flow cytometric analysis of GFP+ cells three days after co-transfection of donor DNA, with or without nucleases as dot plots (forward scatter, FSC vs. GFP). Panel 1: no nuclease showing the background level; Panel 2-3: with spCas9 and either T1 or T2 gRNAs; Panel 4: co-transfection of the NgAgo expression vector; Panel 5-6: NgAgo with either D1 or D2 guide RNAs. **C.** Quantitative data from three duplicates. Data were plotted as mean +/- SD.

Additional materials and methods:

1. Humanized CMV-spCas9 expression vector (9): Addgene #41815;
2. gRNA-AAVS1-T1 (9): Addgene #41817;
3. gRNA-AAVS1-T2 (9): Addgene #41818;
4. CMV-NLS-NgAgo-SK expression vector (8): Addgene #78253;
5. The lentiviral vector EG*IP-AAVS1 reporter (9) is a gift from Dr. Prashant Mali, made by the method described previously (2, 4, 9).

Human 293T cells containing the genomically integrated EG*IP-AAVS1 sequence were made as previously described (2, 9). 15,000 cells per well were plated in a 24-well plate one day before transfection. In addition to 0.4 μg DNA of the t37-GFP donor template, 0.4 μg of spCas9 (or NgAgo) expression vector plus 0.4 μg gRNA template (or guide DNA) were used for co-transfection by lipofectamine 2000 (Invitrogen). Three days after, total cells were harvested and analyzed by a FACS Calibur analyzer as we did before (1-7).

NgAgo failed to induce indels at targeted loci in human cells

Yuexin Zhou, Ping Xu and Wensheng Wei
BIOPIC, ICG, CLS, and School of Life Sciences, Peking University.
Email address: wswei@pku.edu.cn

Summary of finding:
We tested NgAgo's function in human cells and did not observe any indels at CSPG4 locus (Figure below). We also attempted to repeat data from Fig 4 of Gao et al and failed (Fig in correspondence to Editor).

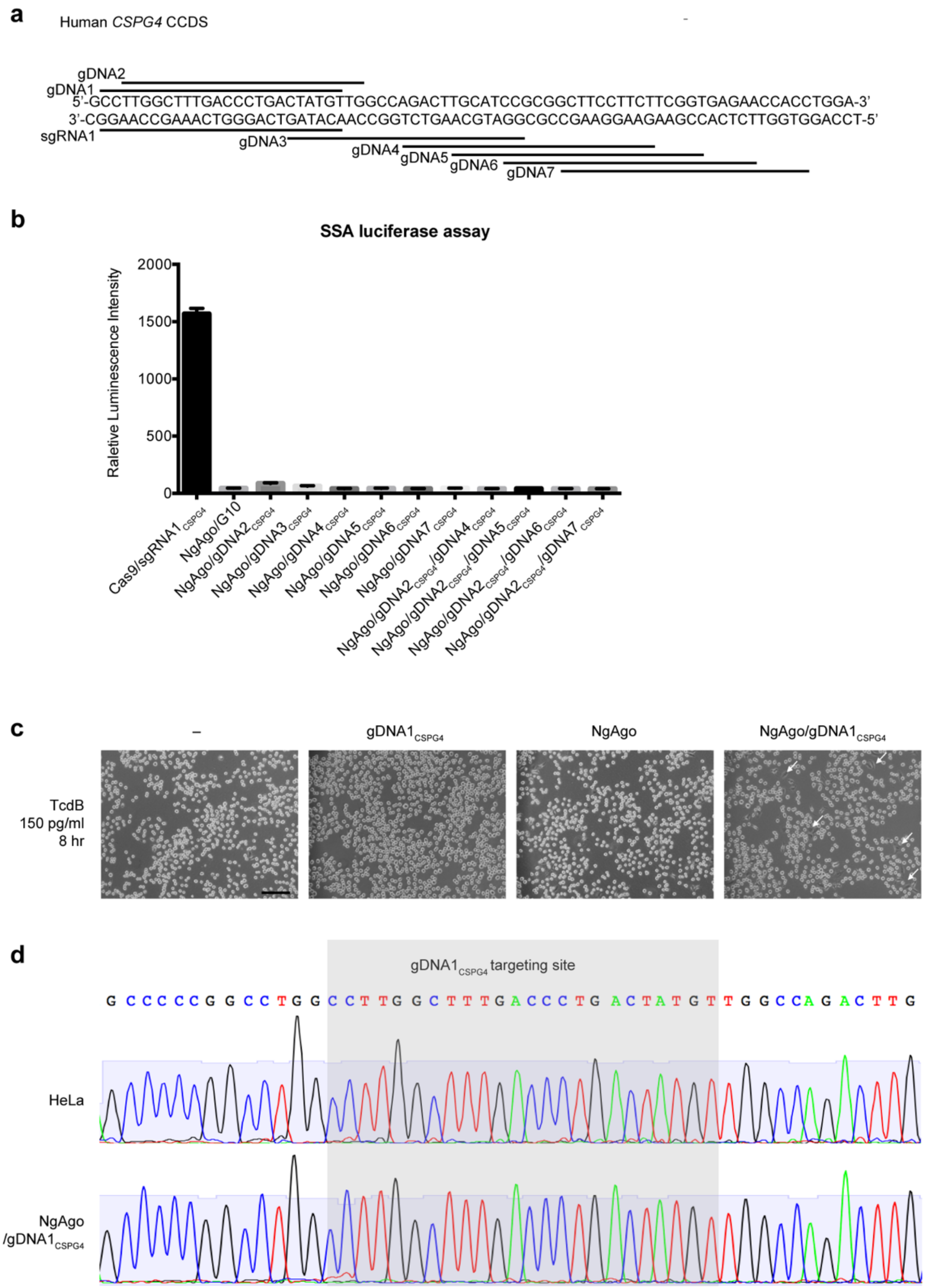

Figure 1. No DSBs were detected when NgAgo system was used to target human *CSPG4* locus. (**a**) Schematic of the different guide DNAs and sgRNA corresponding to the loci of *CSPG4* CCDS sequence. (**b**) SSA-based dual luciferase assay for CRISPR/Cas or NgAgo/gDNA caused DSBs in HEK293T cells. Cells were assayed with Dual-Glo Luciferase Assay 48 hours after transfection. G10, the guide DNA which target *DYRK1A* locus, was used as a negative control. (**c**) Images of different groups of HeLa cells treated with *Clostridium difficile* toxin B (TcdB). HeLa cells were transfected with $gDNA1_{CSPG4}$, NgAgo or NgAgo/$gDNA1_{CSPG4}$. 4 days after transfection, each group of cells were treated with 150 pg/ml TcdB for 8 hr. White arrows indicates some toxin resistant cells. Scale bar, 200 μm. (**d**) Sequencing chromatogram of targeted region of *CSPG4* from wild-type and TcdB resistant cells after NgAgo/$gDNA1_{CSPG4}$ transfection. The sgRNA targeting site are shaded.

Method

Cell culture and plasmid/gDNA construction

HeLa and HEK293T cells were maintained in Dulbecco's modified Eagle's medium (DMEM, Corning) supplemented with 10% fetal bovine serum (FBS, Lanzhou Bailing Biotechnology Co., Ltd.) and 5% CO_2 at 37℃. NgAgo plasmid was a gift from Dr. Han. The gDNAs were individually designed and synthesized with 5' phosphorylation (Ruibiotech).

T7E1 assay of NgAgo targeting *DYRK1A*

3.5×10^5 HEK293T cells were seeded in 6-well plate 24 hr before transfection. The cells were transfected with NgAgo (1 μg)/G10 (500 ng or 1 μg), or NgAgo (1 μg)/G10 (500 ng) followed by the second transfection with G10 (500 ng) in 12 hr. 4 days after first transfection, genomic DNA of different groups of cells were extracted using DNeasy Blood & Tissue Kit (Qiagen) for PCR amplification. PCR products were annealed in NEBuffer 2 (NEB) and then digested with T7 endonuclease 1 (NEB).

2.5% agarose gel electrophoresis was used to analyze the results. Primers used for PCR reaction and T7E1 assay are: 5'-GTTCTTTCAGGTGCGTCA-3' and 5'-GGGACTCTTCTCTATCAGCC-3'.

Single strand annealing (SSA) mediated dual luciferase assay

1.5×10^5 HEK293T cells were seeded in 12-well plate 24 hr before transfection. The cells were co-transfected either with NgAgo, guide DNA, SSA reporter and renilla plasmid (150:150:50:1), or with NgAgo, two guide DNAs, SSA reporter and renilla plasmid (150:75:75:50:1). Cells transfected with Cas9/sgRNA/SSA reporter/renilla plasmid (150:150:50:1) served as positive controls. Cells were assayed with Dual-Glo Luciferase Assay Kit (Promega) 48 hr after transfection according to the manufacture's recommended protocol. G10, the guide DNA which target *DYRK1A* locus, was used as a negative control.

***CSPG4* locus sequencing of TcdB resistant cells**

1.5×10^5 of HeLa cells were seeded at 6-well plate 24 hr before transfection. NgAgo plasmid and gDNA targeting *CSPG4* locus (1 μg each) were co-transfected into HeLa cells by X-tremeGENE HP (Roche). 4 days after transfection, cells were treated with 150 pg/ml TcdB for 8 hr. TcdB resistant cells were kept on culturing for one additional week. Genomic DNA of wild-type and TcdB resistant cells were extracted using DNeasy Blood & Tissue Kit (Qiagen) for PCR amplification of the genome region containing the gDNA targeting sequence. PCR products were subjected for Sanger sequencing. Primers used for PCR reaction and sequencing analysis are:
5'-CTCAGCTCCCAGCTCCCAGGACT-3'and
5'-GCCCCACTGGTGTCTCAGACAATC-3'.

NgAgo failed to induce targeted mutation in 293T cells

Guanghai Xiang[1,2], Wei Mu[1,2], Haoyi Wang[1,2]

[1]State Key Laboratory of Stem cell and Reproductive Biology, Institute of Zoology, Chinese Academy of Sciences, Beijing, China
[2]University of Chinese Academy of Sciences, Beijing, China

Email address: wanghaoyi@ioz.ac.cn

Summary of finding:
To repeat Han's work, we targeted eGFP expressing plasmid and endogenous genes in 293T cells using NgAgo system, following published methods. In all experiments, we failed to detect NgAgo mediated indel mutations at target sites.

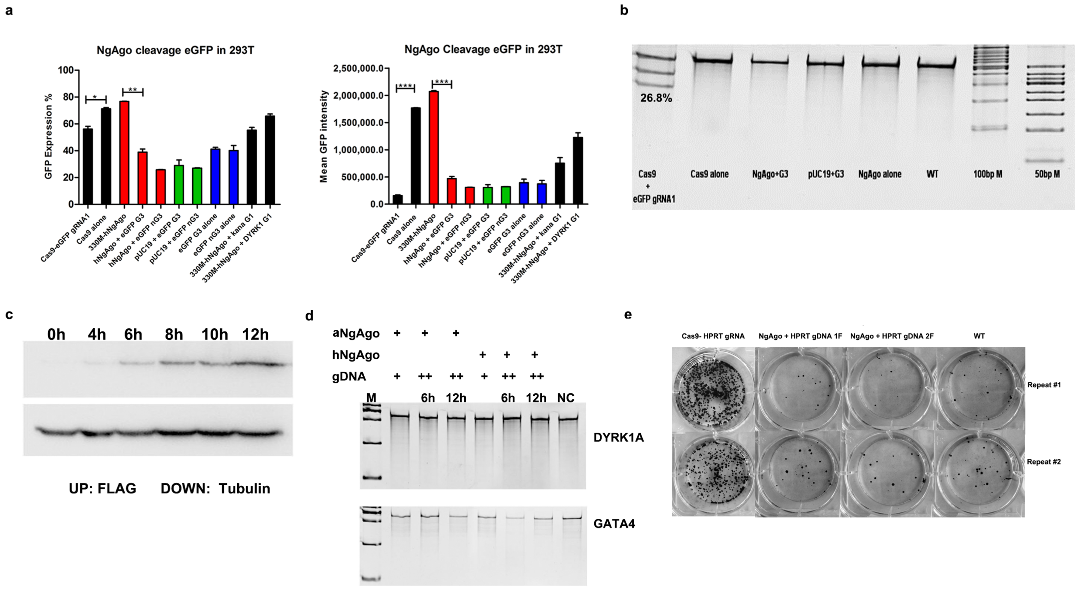

Figure 1. NgAgo failed to cleave DNA targets in culture cells.

(a) Targeting EGFP plasmid in 293T cells. Plasmids expressing *sp*Cas9/sgRNA or NgAgo together with gDNA were co-transfected with pEGFP-N1 plasmid into 293T cells. 2 days after transfection, cells were collected for FACS analysis to determine the percentage of GFP positive cells and mean GFP intensity. 330M-hNgAgo: human codon optimized NgAgo expressed from px330 backbone; eGFP G3: gDNA targeting eGFP as published in Han's paper; nG3: gDNA G3 without 5'-phosphorylation; kana G1: gDNA targeting kanamycin resistance gene on pEGFP-N1 plasmid; DYRK1 G1: gDNA targeting *DYRK1* gene as published in Han's paper. (b) Detect targeted mutation on pEGFP-N1 plasmid using Surveyor Assay. Total DNA was extracted from selected samples in panel a. EGFP target region was amplified by PCR and subjected to Surveyor assay. No indel mutation was detected in NgAgo treated sample. (c) NgAgo expression level at different time points. 6 μg human codon optimized Flag-hNgAgo-expressing plasmid were transfected into 293T cell using lipofectamine2000, the protein level of Flag-hNgAgo was detected by Western Blotting at 0 h, 4 h, 6 h, 8 h, 10 h, and 12h post transfection. (d) NgAgo targeting *DYRK1A* and *GATA4*. 200 ng plasmids expressing either archaea codon NgAgo (aNgAgo) or human codon optimized NgAgo (hNgAgo) were co-transfected with 500 ng *DYRK1A* or *GATA4* gDNA into 293T cells respectively. *DYRK1A* and *GATA4* gDNAs were re-transfected 6 h or 12 h later based on the Western blot result in panel c. Indel mutations at the target sites were evaluated by surveyor assay. NC: non-transfected control. (e) *Sp*Cas9 and hNgAgo were used to target *HPRT1* gene in HCT116 cells. 2 days after transfection, cells were re-plated and selected with 6-TG (10μg/ml) for 10 days, and colonies were stained using Crystal Violet.

Archaea-NgAgo:

**CCAAAGAAGAAGCGGAAGGTCATGACAGTGATTGACCTCGATTCGACCACCACCGCAGACGAACTGACATC
GGGACACACGTACGACATCTCAGTCACGCTCACCGGTGTCTACGATAACACCGACGAGCAGCATCCTCGCATG
TCTCTCGCATTCGAGCAGGACAACGGCGAGCGGCGTTACATTACCCTGTGGAAGAACACGACACCCAAGGAT
GTCTTTACATACGACTACGCCACGGGCTCGACGTACATCTTCACTAACATCGACTACGAAGTGAAGGACGGCT
ACGAGAATCTGACTGCAACATACCAGACGACCGTCGAGAACGCTACCGCTCAGGAAGTCGGGACGACTGAC
GAGGACGAAACGTTCGCGGGCGGCGAGCCGCTCGACCATCACTTGGACGACGCGCTCAATGAGACGCCAGA
CGACGCGGAGACAGAGAGCGACTCAGGCCATGTGATGACCTCGTTCGCCTCCCGCGACCAACTCCCTGAGTG
GACGCTGCATACGTATACGCTAACAGCCACAGACGGCGCAAAGACGGACACGGAGTACGCGCGACGAACCC
TCGCATACACGGTACGGCAGGAACTCTATACCGACCATGATGCGGCTCCGGTTGCAACTGACGGGCTAATGC
TTCTCACGCCAGAGCCGCTCGGCGAGACCCCGCTTGACCTCGATTGCGGTGTCCGGGTCGAGGCGGACGAGA
CTCGGACACTCGATTACACCACGGCCAAAGACCGGTTACTCGCCCGCGAACTCGTCGAAGAGGGGCTCAAAC
GCTCCCTCTGGGATGACTACCTCGTTCGCGGCATCGATGAAGTCCTCTCAAAGGAGCCTGTGCTGACTTGCGA
TGAGTTCGACCTACATGAGCGGTATGACCTCTCTGTCGAAGTCGGTCACAGTGGGCGGGCGTACCTTCACATC
AACTTCCGCCACCGGTTCGTACCGAAGCTGACGCTCGCAGACATCGATGATGACAACATCTATCCTGGGCTCC
GGGTGAAGACGACGTATCGCCCCCGGCGAGGACATATCGTCTGGGGTCTGCGGGACGAGTGCGCCACCGAC
TCGCTCAACACGCTGGGAAACCAGTCCGTCGTTGCATACCACCGCAACAATCAGACACCTATTAACACTGACC
TCCTCGACGCTATCGAGGCCGCTGACCGGCGAGTCGTCGAAACCCGACGTCAAGGGCACGGCGATGATGCTG
TCTCATTCCCCCAAGAACTGCTTGCGGTCGAACCGAATACGCACCAAATTAAGCAGTTCGCCTCCGACGGATT
CCACCAACAGGCCCGCTCAAAGACGCGTCTCTCGGCCTCCCGCTGCAGCGAGAAAGCGCAAGCGTTCGCCGA
GCGGCTTGACCCGGTGCGTCTCAATGGGTCCACGGTAGAGTTCTCCTCGGAGTTTTTCACCGGGAACAACGA
GCAGCAACTGCGCCTCCTCTACGAGAACGGTGAGTCGGTTCTGACGTTCCGCGACGGGGCGCGTGGTGCGCA
CCCCGACGAGACATTCTCGAAAGGTATCGTCAATCCACCAGAGTCGTTCGAGGTGGCCGTAGTACTGCCCGA
GCAGCAGGCAGATACCTGCAAAGCGCAGTGGGACACGATGGCTGACCTCCTCAACCAAGCTGGCGCGCCACC
GACACGGAGCGAGACCGTCCAATATGATGCGTTCTCCTCGCCAGAGAGCATCAGCCTCAATGTGGCTGGAGC
CATCGACCCTAGCGAGGTAGACGCGGCATTCGTCGTACTGCCGCCGGACCAAGAAGGATTCGCAGACCTCGC
CAGTCCGACAGAGACGTACGACGAGCTGAAGAAGGCGCTTGCCAACATGGGCATTTACAGCCAGATGGCGT
ACTTCGACCGGTTCCGCGACGCGAAAATATTCTATACTCGTAACGTGGCACTCGGGCTGCTGGCAGCCGCTGG
CGGCGTCGCATTCACAACCGAACATGCGATGCCTGGGGACGCAGATATGTTCATTGGGATTGATGTCTCTCG
GAGCTACCCCGAGGACGGTGCCAGCGGCCAGATAAACATTGCCGCGACGGCGACCGCCGTCTACAAGGATG
GAACTATCCTCGGCCACTCGTCCACCCGACCGCAGCTCGGGGAGAAACTACAGTCGACGGATGTTCGTGACA
TTATGAAGAATGCCATCCTCGGCTACCAGCAGGTGACCGGTGAGTCGCCGACCCATATCGTCATCCACCGTGA
TGGCTTCATGAACGAAGACCTCGACCCCGCCACGGAATTCCTCAACGAACAAGGCGTCGAGTACGACATCGT
CGAAATCCGCAAGCAGCCCCAGACACGCCTGCTGGCAGTCTCCGATGTGCAGTACGATACGCCTGTGAAGAG
CATCGCCGCTATCAACCAGAACGAGCCACGGGCAACGGTCGCCACCTTCGGCGCACCCGAATACTTAGCGAC
ACGCGATGGAGGCGGCCTTCCCCGCCCAATCCAAATTGAACGAGTCGCCGGCGAAACCGACATCGAGACGCT
CACTCGCCAAGTCTATCTGCTCTCCCAGTCGCATATCCAGGTCCATAACTCGACTGCGCGCCTACCCATCACCA
CCGCATACGCCGACCAGGCAAGTACTCACGCGACCAAGGGTTACCTCGTCCAGACCGGAGCGTTCGAGTCTA
ATGTCGGATTCCTCTAA**

NLS

Bac-NgAgo

Stop codon

Humanized NgAgo

ATGGACTATAAGGACCACGACGGAGACTACAAGGATCATGATATTGATTACAAAGACGATGACGATAAGAT
GGCCCCAAAGAAGAAGCGGAAGGTCGGTATCCACGGAGTCCCAGCAGCCATGACCGTCATCGATCTTGACA
GTACCACTACAGCAGATGAGCTTACTTCAGGCCATACCTACGATATCTCAGTCACCTTGACAGGGGTCTATGA
TAATACTGATGAGCAGCACCCGCGGATGAGTCTGGCGTTCGAGCAAGATAACGGTGAGCGAAGGTACATAA
CTCTGTGGAAAAATACAACGCCTAAAGACGTATTTACGTACGACTACGCAACCGGCTCCACCTATATCTTCAC
CAACATAGACTATGAGGTTAAGGATGGTTACGAGAATCTCACAGCTACGTATCAAACGACTGTTGAAAATGC
GACTGCTCAAGAGGTGGGAACCACGGATGAAGACGAAACATTCGCCGGAGGGGAGCCGCTTGATCACCACC
TTGATGACGCACTGAACGAAACACCAGATGATGCCGAGACGGAGTCTGACTCCGGGCACGTAATGACGTCTT
TCGCTTCTCGAGATCAACTGCCGGAGTGGACCCTCCATACGTATACTCTTACCGCAACTGATGGAGCCAAAAC
TGATACAGAATACGCGAGGCGAACGCTGGCTTATACTGTTCGCCAAGAATTGTATACGGATCATGATGCAGC
TCCGGTCGCAACAGACGGGCTTATGCTTCTCACCCCTGAGCCATTGGGGGAGACACCACTCGACCTGGACTGC
GGTGTAAGAGTTGAGGCGGATGAAACGAGGACATTGGATTATACGACAGCAAAGGATAGGCTTCTCGCTCG
AGAGCTGGTCGAGGAGGGCCTGAAAAGGTCCCTCTGGGATGACTACCTGGTTAGGGGAATCGACGAAGTGT
TGTCTAAGGAACCCGTTCTTACTTGCGATGAGTTTGATTTGCACGAGCGCTACGATCTCTCCGTGGAAGTAGG
GCATTCTGGTCGGGCCTACTTGCACATCAATTTTAGACACAGATTCGTCCCAAAGTTGACATTGGCAGATATC
GATGATGATAATATATATCCAGGTCTCAGGGTGAAAACCACCTACAGGCCAAGGCGCGGTCATATCGTCTGG
GGCCTTCGCGACGAGTGCGCCACAGATTCATTGAACACCTTGGGAAATCAGAGCGTTGTGGCGTACCACCGG
AATAACCAAACCCCGATTAACACTGACCTTTTGGATGCGATTGAAGCCGCCGATCGACGGGTTGTGGAGACG
CGAAGACAAGGGCATGGAGACGATGCTGTGTCCTTTCCACAAGAGCTCCTGGCGGTAGAGCCGAACACTCAC
CAGATTAAACAATTCGCGAGTGATGGCTTTCACCAACAAGCGCGAAGCAAAACGCGGCTTTCCGCGTCTAGG
TGTTCAGAGAAAGCCCAAGCGTTCGCAGAGAGGCTGGACCCCGTCCGGCTGAATGGGTCAACTGTTGAATTC
AGTTCAGAATTCTTCACTGGCAACAACGAACAGCAACTTAGGTTGCTCTATGAAAACGGAGAAAGTGTACTC
ACATTTCGCGATGGAGCGCGCGGTGCCCATCCAGACGAGACGTTCAGTAAGGGTATCGTTAATCCGCCAGAA
AGCTTCGAAGTAGCAGTCGTTCTGCCCGAGCAACAGGCGGACACCTGCAAAGCACAATGGGATACCATGGCC
GACCTGCTTAATCAAGCCGGGGCACCCCCGACGAGAAGCGAAACAGTACAGTATGACGCGTTCTCCAGTCCC
GAAAGTATATCCCTGAATGTGGCGGGTGCAATAGACCCTTCAGAGGTAGATGCCGCGTTCGTCGTACTGCCTC
CTGATCAAGAAGGGTTTGCGGATCTGGCGAGCCCGACCGAGACTTACGATGAACTCAAGAAAGCGCTCGCA
AACATGGGCATCTATAGTCAAATGGCGTACTTTGACAGGTTCAGGGACGCGAAAATCTTTTATACACGCAAC
GTTGCACTCGGGTTGTTGGCTGCTGCAGGCGGGGTGGCTTTTACAACAGAGCACGCGATGCCTGGAGATGCA
GATATGTTTATCGGGATAGATGTATCACGCTCTTATCCAGAGGACGGAGCGTCTGGGCAGATTAACATAGCC
GCTACGGCTACAGCTGTATATAAGGATGGTACCATTCTGGGACATTCAAGCACTCGGCCACAGCTCGGTGAG
AAGTTGCAAAGTACGGATGTGCGGGACATTATGAAAAACGCAATTCTTGGTTACCAGCAAGTAACGGGCGA
AAGTCCGACCCATATTGTGATACATCGAGACGGCTTTATGAACGAGGATCTTGATCCCGCTACTGAATTCCTC
AACGAACAAGGTGTGGAGTACGACATTGTGGAAATCCGGAAGCAGCCCCAAACTAGACTGCTCGCGGTATC
AGACGTCCAATACGATACGCCCGTGAAGAGTATTGCAGCTATAAATCAAAACGAGCCCCGCGCCACGGTTGC
AACATTTGGGGCGCCAGAGTATTTGGCGACCCGGGATGGCGGGGGATTGCCCCGACCAATTCAAATCGAAC
GAGTAGCTGGTGAGACTGATATTGAGACGCTCACGCGACAGGTGTACCTGCTTAGCCAATCCCACATTCAGG
TCCATAATTCTACAGCTCGATTGCCCATCACTACCGCTTACGCAGATCAAGCTAGCACTCATGCGACTAAGGGC
TACTTGGTTCAAACTGGGGCTTTCGAATCTAATGTAGGATTCCTGTAA

SV40 NLS + 3 X Flag

Humanized NgAgo

Stop codon

gDNA sequences:

eGFP nG3 : 5’AAGGGCGAGGAGCTGTTCACCGGG

eGFP G3 : 5’p-AAGGGCGAGGAGCTGTTCACCGGG

DYRK1A G10: 5’p-CCAAAGTCCAAGGTATTAGCAGCC

GATA4 G41: 5’p-AGCTCCGGTGGGGCCGCGTCTGGT

Kana G1: 5’p-GGATCGTTTCGCATGATTGAACAA

HPRT G1F: 5’p-TTGCTGGTGAAAAGGACCCCACGA

HPRT G2F: 5’p-TATAAGCCAGACTGTAAGTGAATT

sgRNA sequences:

eGFP sgRNA (20nt) : 5’-gggcacgggcagcttgccgg-3’

HPRT sgRNA (20nt): 5’-gaaagtaattcacttacagtc-3’

NgAgo failed to cleave DNA targets in culture cells and mouse embryos

Changyang Zhou[1], Hui Yang[1]

[1]Institute of Neuroscience, State Key Laboratory of Neuroscience, Key Laboratory of Primate Neurobiology, CAS Center for Excellence in Brain Science and Intelligence Technology, Shanghai Institutes for Biological Sciences, Chinese Academy of Sciences, Shanghai 200031, China

Email address: huiyang@ion.ac.cn (H.Y.).

Summary of finding:
We performed experiments as described in Fig legend and Fig 1. We did not observe any NgAgo mediated genome editing in cultured cells and mouse embryos.

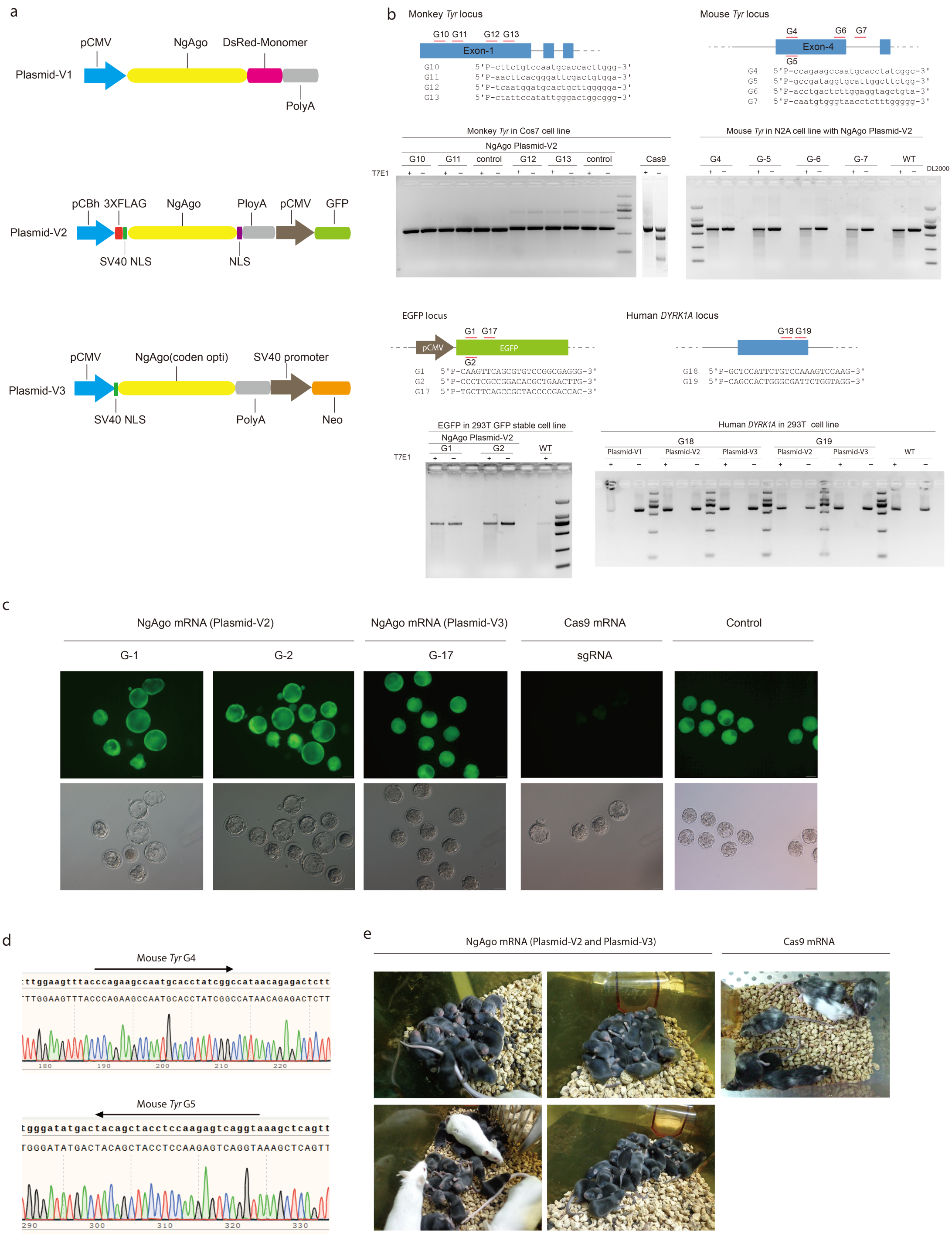

f

NgAgo-mediated gene editing in mouse embryos				
NgAgo mRNA (ng/ul)	gDNA (ng/ul)	Embryo transferred (No.)	Newborn mice (No.)	*Tyr*-knockout mice (No.)
NgAgo (V2) (50)	*Tyr*-gDNA (20)	265	131	0
NgAgo (V2) (100)	*Tyr*-gDNA (50)	372	199	0
NgAgo (V3) (100)	*Tyr*-gDNA (50)	158	70	0

Figure 1. NgAgo failed to cleave DNA targets in culture cells and mouse embryos. (a) Schematic diagram of the three versions of the NgAgo vector. (b) Schematic of the guides corresponding to the loci of each gene (top) and T7E1 assay (bottom) for NgAgo-mediated gene editing. Different cultured cells (Cos7 cell line, 293T cell line and N2A cell line) were transfected with NgAgo and corresponding gDNAs. Two days later, the genomic DNA was isolated and performed T7E1 assay. The positive control was genomic DNA isolated from Cos7 cells transfected with CRISPR/Cas9 system. No cutting band could be observed in the cells treated with NgAgo-gDNA system. (c) Images of GFP signal of blastocysts resulting from different forms of GFP targeting. NgAgo mRNA and gDNAs targeting GFP were injected into individual mouse zygotes and GFP signal was examined at blastocyst stage. As a control, Cas9 mRNA and single sgRNA targeting GFP were injected into individual mouse zygotes and GFP signal was examined at blastocyst stage. (d) DNA sequences of blastocsyt resulting from *Tyr*-NgAgo targeting. (e) Representative results of pigmentation phenotypes of mice resulting from *Tyr* targeting. NgAgo mRNA and gDNAs targeting *Tyr* were injected into individual mouse zygotes and the injected zygotes were then transferred into recipients. As a control, Cas9 mRNA and single sgRNA targeting *Tyr* were injected. Mice carrying one or two wild-type copies of *Tyr* are fully pigmented, whereas mice that are homozygous for a null allele are albino. Therefore, genetic mosaicism could be assessed by visual inspection. (f) NgAgo-mediated gene editing in mouse embryos。

NgAgo failed to cleave DNA targets in culture cells and mouse embryos

Xiukun Wang[1], Jinsong Li[1]

[1]State Key Laboratory of Cell Biology, CAS Center for Excellence in Molecular Cell Science, Institute of Biochemistry and Cell Biology, Shanghai Institutes for Biological Sciences, Chinese Academy of Science, Shanghai, 200031, China
Email address: jsli@sibcb.ac.cn

Summary of finding:
We performed experiments as described in Figs 1-3. We did not observe any NgAgo mediated genome editing in cultured cells and mouse embryos.

Fig. 1

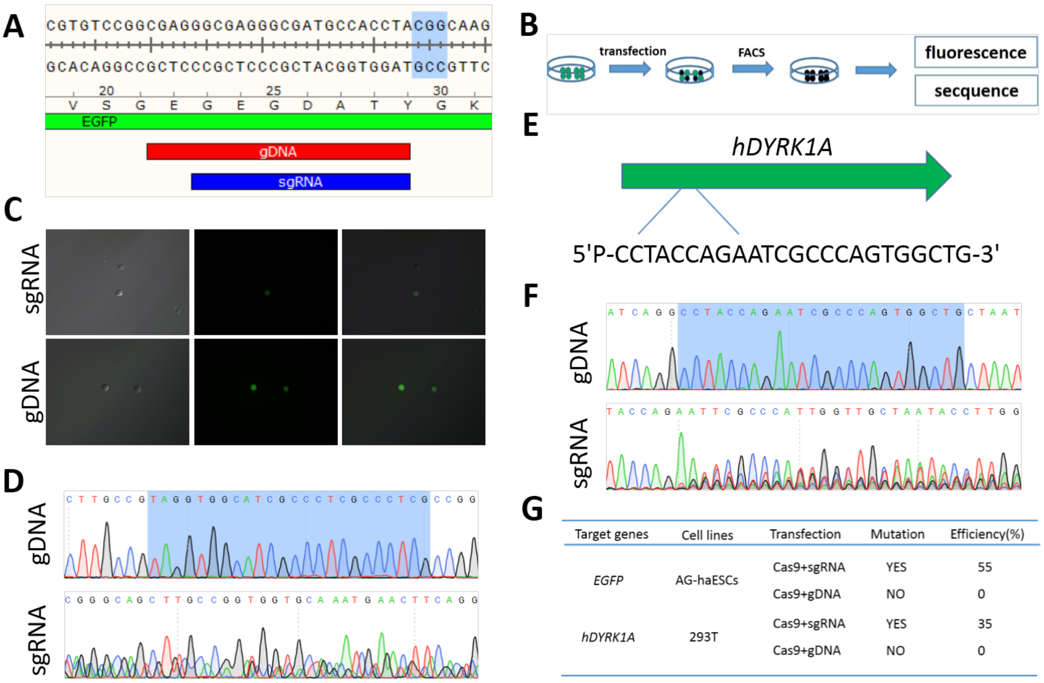

Target genes	Cell lines	Transfection	Mutation	Efficiency(%)
EGFP	AG-haESCs	Cas9+sgRNA	YES	55
		Cas9+gDNA	NO	0
hDYRK1A	293T	Cas9+sgRNA	YES	35
		Cas9+gDNA	NO	0

Fig 1. NgAgo fails to mutate genes in androgenetic haploid embryonic stem cells (AG-haESCs) and 293T cells.

(A) The sequence of sgRNA and gDNA targeting in EGFP coding sequence. (B) Diagram of targeting EGFP gene in AG-haESCs and hDYRK1A gene in 293T cells. (C) The fluorescence of AG-haESCs transfecting Cas9/sgRNA and NgAgo/gDNA after FACS. (D) The sequence of targeted EGFP gene via Cas9/sgRNA and NgAgo/gDNA in AG-haESC clones. (E) The gDNA-targeting sequence in hDYR1A gene. (F) The sequence of targeting hDYRK1A gene via Cas9/sgRNA and NgAgo/gDNA in 293T cells. (G) Summary of mutation efficiency induced by CRISPR-Cas9 and NgAgo.

Fig. 2

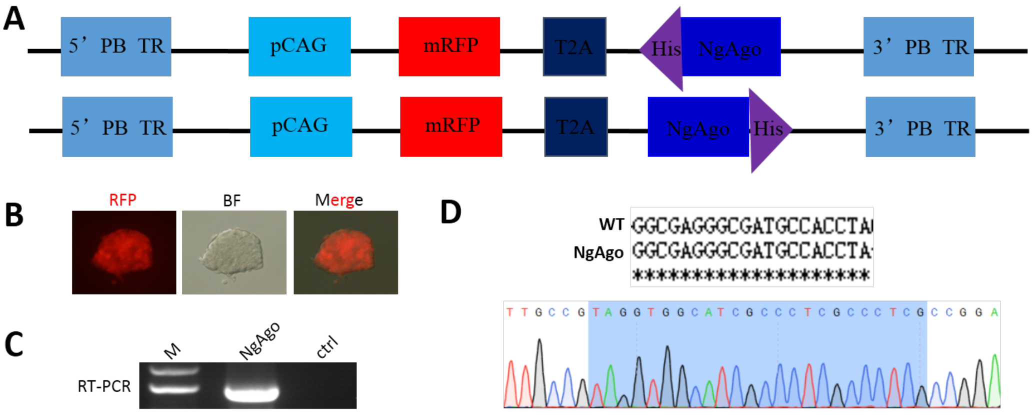

E

Target genes	Cell lines	Transfection	Mutation	Efficiency(%)
EGFP	AG-haESCs	gDNA	NO	0

Fig 2. AG-haESCs stably expressing NgAgo fails to mutate gene.
(A) Strategy of constructing plasmids expressing NgAgo protein stably. (B) Generation of AG-haESCs stably expressing NgAgo protein indicated by red fluorescent protein expression. (C) RT-PCR analysis of NgAgo expression in haploid cells. (D) The sequence of gDNA used for mutagenesis in AG-haESCs carrying constitutive expression of NaAgo. (E) No mutant cells were observed in experiments.

Fig. 3

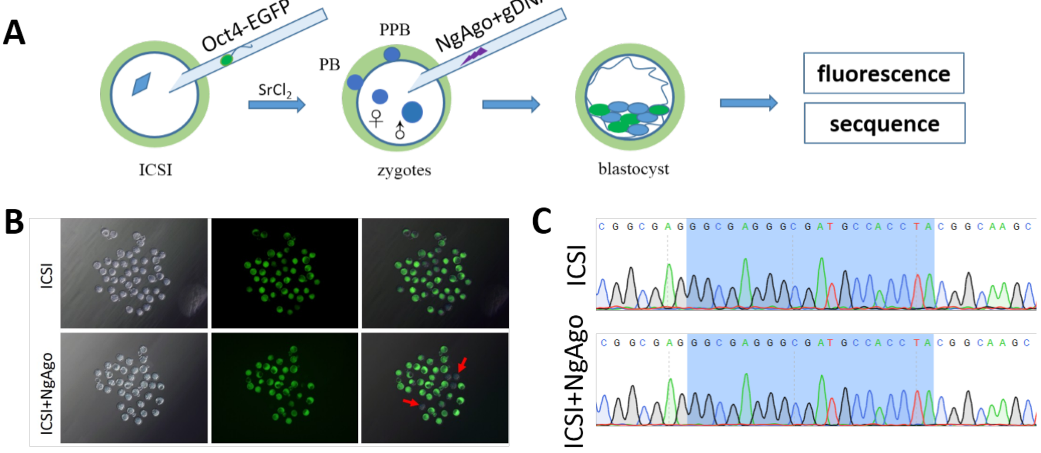

D

Target genes	no fluorescence/total embryos	NgAgo+gDNA(ng)	mutation
ctrl	0/47	50+0	NO
EGFP	6/114	50+50	NO
EGFP	4/97	100+50	NO

Fig 3. NgAgo fails to mutate EGFP transgene in mouse embryos.
(A) Schematic of NgAgo-mediated gene mutation in mouse embryos. (B) Fluorescent analysis of blastocysts developed after injection of NgAgo/gDNA into zygotes. (C) Sequencing analysis of EGFP transgene in E3.5 blastocyst. (D) Summary of NgAgo-mediated mutation in mouse embryos.

NgAgo failed to label telomeres in living human cells

Shipeng Shao[1], Yujie Sun[1]

[1]State Key Laboratory of Membrane Biology, Biodynamic Optical Imaging Center (BIOPIC), School of Life Sciences, Peking University, Beijing 100871, China.

Email address: sun_yujie@pku.edu.cn (Y.S.).

Summary of finding:
We performed experiments to label telomeres in living human cells using fluorescent NgAgo as described in Fig legend and Fig 1. We did not observe specific binding of NgAgo to telomeres with the aid of gDNA that targets the repeat sequence of telomeres.

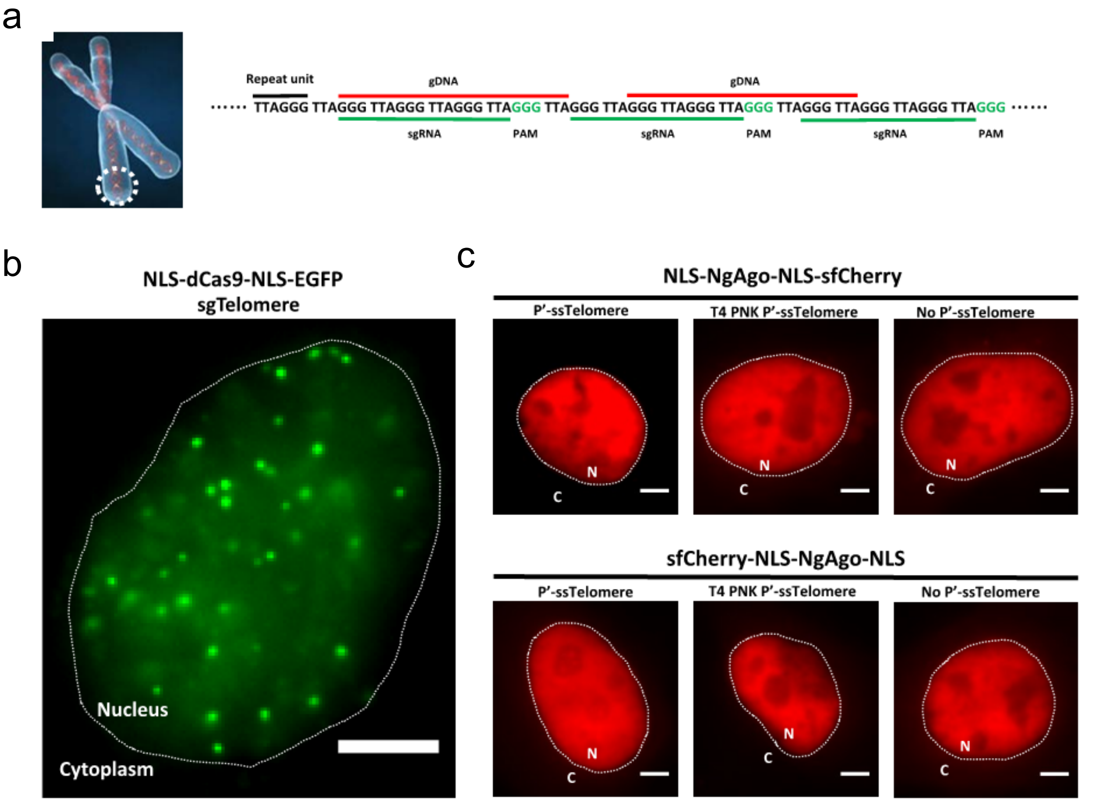

Figure 1. Telomere labeling and imaging by dCas9 and NgAgo in living human cells. (a) sgRNA and gDNA design diagram for telomere labeling. Telomeres, specialized structures which localize at both ends of each chromosome, consist of multiple 'TTAGGG' repeats of a short tract about 5 to 15 kb. Such repeats allow the recruitment of multiple fluorescent protein-fused dCas9 or NgAgo molecules to the same locus using a single sgRNA or gDNA sequence, which renders a direct

readout for detection of whether dCas9 or NgAgo can bind DNA. Successful binding can generate fluorescent puncta while no interaction between proteins and DNA will result in a diffusive distribution of the fluorescent proteins. Green lines represent the sgRNA binding sites and red lines indicate the gDNA binding sites.

(b) Telomere labeling by the CRISPR imaging system. In order to increase the nuclear importing efficiency of the dCas9 protein, two nuclear localization sequences (NLS) were added to both N- and C- termini. NLS-dCas9-NLS was fused with EGFP. Human breast cancer cell line MDA-MB-231 was transfected with 1 µg dCas9-EGFP plasmid and 1µg sgRNA plasmid targeting telomeres. 24h post transfection, live cell imaging was implemented using a Nikon TiE inverted microscope equipped with a 100X oil objective. Multiple fluorescent puncta were visualized in the nucleus, suggesting that dCas9 proteins bind telomeres in the presence of sgRNA. White line indicates the boundary of the cell nucleus. (c) Telomere labeling by the NgAgo imaging system. In order to increase the nuclear importing efficiency of the NgAgo protein, two nuclear localization sequences (NLS) were added to both N- and C- termini. NLS-NgAgo-NLS was fused with super fold Cherry (sfCherry). Cells were transfected with 1µg NgAgo-sfCherry (or sfCherry-NgAgo) plasmid and 1µg gDNA (5' phosphorylated by commercial synthesis, T4 PNK or without phosphorylation) targeting telomeres. 24h post transfection, live cell imaging was carried out with the same condition as used in CRISPR imaging. In all 6 scenarios, NgAgo showed diffusive distribution in the nucleus, which suggests that NgAgo is unable to bind genome DNA or interact with DNA with an extremely low affinity beyond the detection threshold of the live cell imaging assay. White line indicates the boundary of the cell nucleus. N: Nucleus, C: cytoplasm, Scale bar, 5µm.

NgAgo failed to edit DNA fragments in cultured human cells and mouse embryos

Xiao Ge[1], Yonghu Wu[1], Zhilian Jia[1], Qiang Wu[1]

[1]Center for Comparative Biomedicine, Institute of Systems Biomedicine, MOE Key Laboratory of Systems Biomedicine, Shanghai Jiao Tong University, 800 Dongchuan Road, Minhang district, Shanghai 200140, China

Email address: qwu123@gmail.com (Q.W.).

Summary of finding:
We performed experiments as described in Fig 1. We did not observe any NgAgo mediated DNA fragment editing in cultured cells and mouse embryos.

We developed a CRISPR-based DNA-fragment editing method to investigate the architectural mechanism of the mammalian three-dimensional (3D) genomes. In conjunction with chromosome conformation capture and bioinformatics methods, we found that the location and relative orientation of CTCF-binding sites in enhancers and promoters determines the directionality of chromatin looping and gene expression, revealing a genome-wide general principle underlying the 3D genome architecture and orientation-dependent enhancer function (Cell, 162: 900-910, 2015; J. Mol. Cell Biol. 7: 284-298, 2015). We tried many experiments of DNA fragment editing in human and mouse cell lines as well as mouse embryos, using NgAgo with Cas9 as a control and did not find any edited DNA fragment. An example of results from our experiments is shown in Figure 1.

Cell culture and transfection

293T cells were cultured with DMEM supplemented with 10% FBS.

NgAgo (codon-optimized) expression plasmid (final concentration of 100 ng/ul) was transfected with two phosphorylated gDNAs, targeting two sequences flanking the HS5-1b CTCF-binding site in the human protocadherin enhancer. NgAgo and gDNAs were transfected again 24hr after transfection. Cells were harvested 24hr after the second transfection and DNA was extracted for PCR experiments. As a positive control, we designed two sgRNAs. Cas9 plasmid was transfected at the same condition as a control.

gDNA sequence (for NgAgo):

HS5-1b-gDNA1: 5’-p-CAGTTCGGTCCCATGACTCGCTGC-3’

HS5-1b-gDNA2: 5’-p-GTGTAAAGGGTTCTTTGGATTAAG-3’

gRNA sequence (for Cas9):

HS5-1b-gRNA1: GCTTCCGGTAGGGCGGGGTCGGG

HS5-1b-gRNA2: AGATTTGGGGCGTCAGGAAGTGG

PCR primer:

HS5-1b-F1: CCCTCCACCTCTGGCATTG

HS5-1b-R1: TTTTTGGCTAACAACATAGTGCTTC

(F1+R1: WT 603 bp, Cas9 del 464 bp, NgAgo del 397~445 bp)

HS5-1b-F2: TCCCAAAACAAGGGGTTCCTAC

HS5-1b-R2: AGTAGAAGCGAGAGATCACTCTG

(F2+R2:WT 447 bp, Cas9 del 308 bp, NgAgo del 241~289 bp)

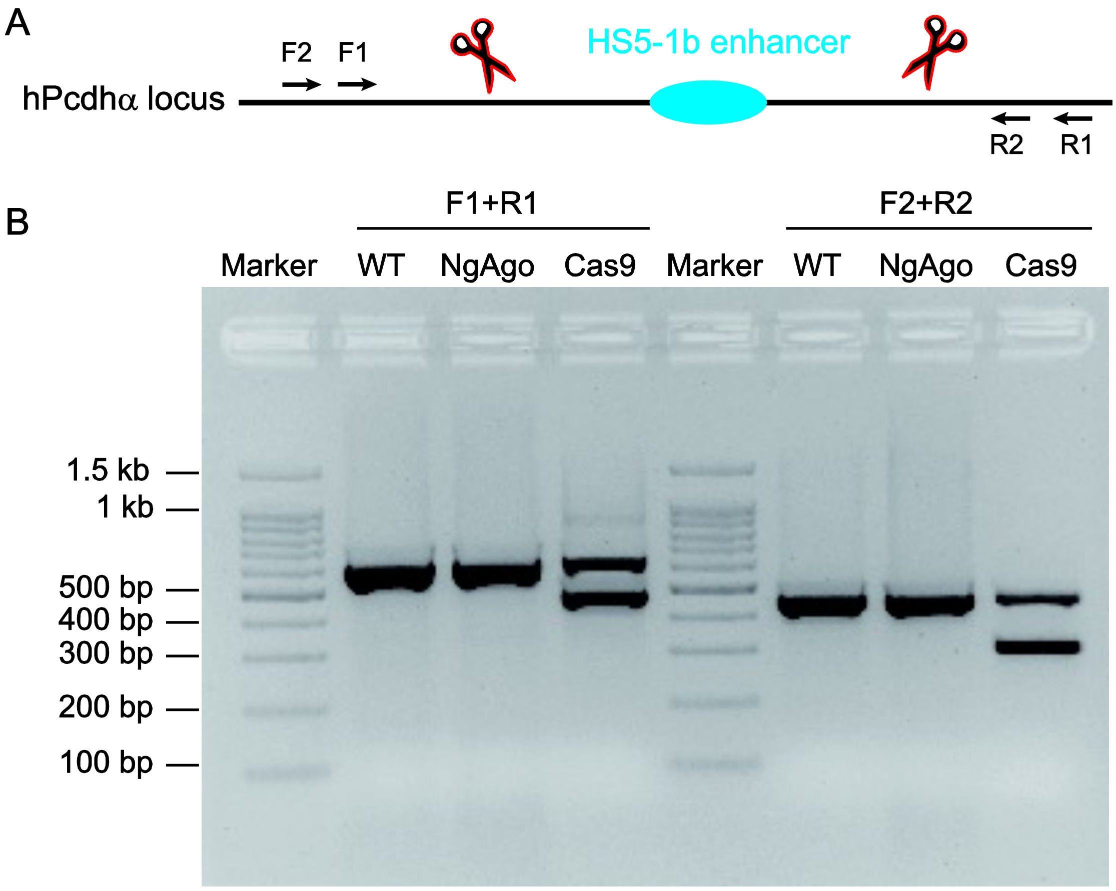

Figure legend

DNA-fragment editing with a pair of guiding sequences

A. Schema of DNA-fragment editing experiments on a human protocadherin (hPcdhα) enhancer, with the cutting sites and PCR primers indicated.

B. PCR gel with the edited genomic DNA as templates. The left and right panel are amplified with different pairs of primers.

NgAgo failed to induce indels in zebrafish genome

Wei Qin
School of Chemical Biology and Biotechnology
Peking University Shenzhen Graduate School
Lishui Road, Xili Town, Nanshan District, Shenzhen
E-mail: qinwei@pkusz.edu.cn

Shuo Lin
Department of Molecular, Cell and Developmental Biology
University of California, Los Angeles
621 Charles E. Young Drive South, CA 90095-1606
E-mail: shuolin@ucla.edu

Summary of finding:
Since pigmentation defect is a convenient phenotype indication for mutation in the tyrosinase (tyr) gene (1-2), which encodes an enzyme that converts tyrosine into melanin, we designed three different gDNA targeting *tyr*. After co-injection of tyr gDNA and NgAgo mRNA into one-cell-stage zebrafish embryos, no difference of pigmentation was observed compared to control group (N >100). We randomly selected multiple embryos, extracted genomic DNA and analyzed indels at the targeted sites using T7E1 and no genome variation was detected. In positive control experiments using tyr gRNA and Cas9 mRNA, pigmentation reduction was clearly observed in embryos (N >100) and some of which totally lacked pigmentation, as previously reported (1-2). In addition, we analyzed multiple genomic loci of zebrafish *mib-1, twist1a*, UROD and GFP transgene using transgenic zebrafish. Again, no genomic indels were detected by NgAgo targeting.

Table: gDNA sequences used to target zebrafish tyr genomic loci.

Gene name	Target sites
Tyr-1	5'p-CCAGAAGTCCTCCAGTCCAAACGC-3'
Tyr-2	5'p-CAACTGCGCAGAGAGACGCGAGTC-3'
Tyr-3	5'p-CTGTCCAGTCTGGCCCGGCGACGG-3'

1. Feng Y, Chen C, Han Y, Chen Z, Lu X, Liang F, Li S, Qin W, Lin S. (2016) Expanding CRISPR/Cas9 Genome Editing Capacity in Zebrafish Using SaCas9. G3 (Bethesda). 6(8):2517-21.
2. Qin W, Liang F, Feng Y, Bai H, Yan R, Li S, Lin S. (2015) Expansion of CRISPR/Cas9 genome targeting sites in zebrafish by Csy4-based RNA processing. Cell Res. 25(9):1074-7.

NgAgo failed to cleave mouse genomic DNA

Hui Zhang[1], Ruilin Sun[2], Yu Zhou[2], Jinjin Wang[2], Bin Zhou[1]
[1]The State Key Laboratory of Cell Biology, Institute of Biochemistry and Cell Biology, Shanghai Institutes for Biological Sciences, Chinese Academy of Sciences, Shanghai 200031, China.
[2]Shanghai Research Center for Model Organisms, Shanghai 201203, China.

Email address: zhoubin@sibs.ac.cn (B.Z.)

Summary of finding:
We did not detect any NgAgo mediated activity in mouse genomic DNA editing (Figure 1 and Figure legend).

Figure 1

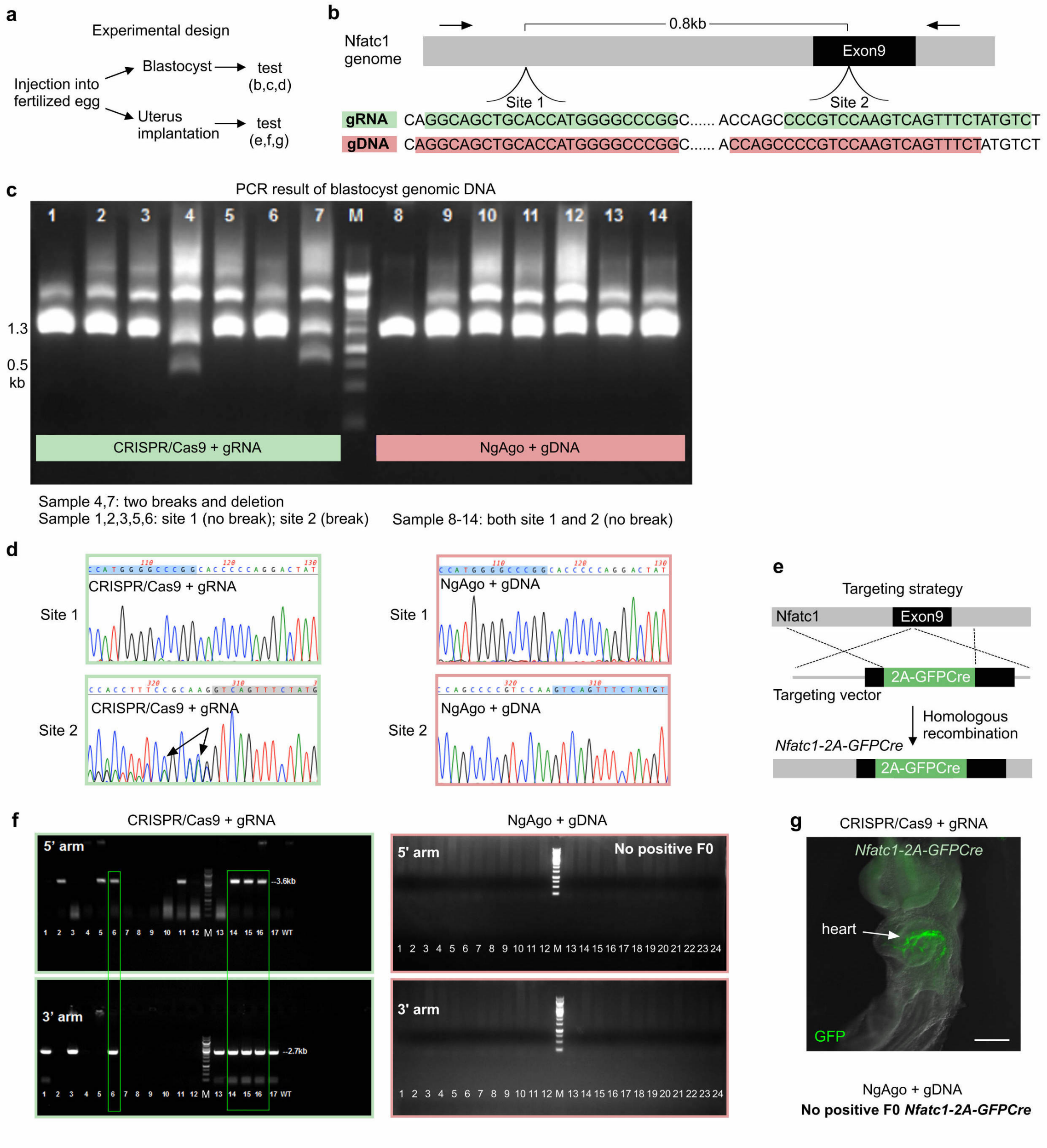

Figure legend

Assessment of NgAgo-mediated Non-homologous end joining (NHEJ) and homologous recombination (HR) on mouse genome editing, in comparison with CRISPR/Cas9.

(a) Schematic figure showing experimental design.

(**b-d**): Detection of CRSPR/Cas9- or NgAgo-mediated nuclease activity in the murine Nfatc1 gene at the blastocyst stage. Cas9 mRNA/two gRNAs or NgAgo mRNA/two gDNAs were mixed and microinjected into the cytoplasm of zygotes. The injected zygotes were cultured until blastocyst stage by 3.5 days. (**b**) Schematic figure showing *Nfatc1* gRNAs (green) or gDNAs (red) targeting intron 8 and exon 9 in the murine *Nfatc1* gene. Two arrows indicate the location of PCR primers. (**c**) Agarose gel electrophoresis of Nest-PCR products. Lanes 1-7: CRSPR/Cas9-mediated, Lanes 8-14: NgAgo-mediated, M, molecule weight marker. The PCR fragment of wide type allele is 1.3kb. The PCR fragment of mutant allele is about 0.5kb. Lane 4 and 7 were the representative samples with the targeting sequence deletion. (**d**) The representative sequencing results for the PCR products shown in lanes 1-3, 5-6 and 8-14 of (c). Arrows point to dual peaks of DNA sequencing, indicating NHEJ repair after CRISPR/Cas9-mediated DNA break. No dual peaks were observed in NgAgo treated samples.

(**e-g**): Generation of *Nfatc1-2A-GFPCre* mouse model by CRISPR/Cas9 or NgAgo strategies. Cas9 mRNA/gRNA with donor plasmid vector or NgAgo mRNA/gDNA with donor plasmid vector were mixed and microinjected into the pronucleus of zygotes. The injected zygotes were transferred into uterus of pseudo-pregnant female mice. (**e**) Schema of CRISPR/Cas9 or NgAgo-mediated HR in the murine *Nfatc1* gene to generate a *Nfatc1-2A-GFPCre* mouse model. (**f**) Detection of the HR in the CRISPR/Cas9 injected and NgAgo injected mouse embryos by PCR. The PCR product of 5' positive homologous recombination is 3.6kb and the PCR product of 3' positive homologous recombination is 2.7kb. Green boxed samples indicate both 5' and 3' correct targeting. (**g**) Whole-mount GFP fluorescence image of E8.5 positive *Nfatc1-2A-GFPCre* embryo showed specific GFP/Nfatc1 expression in the heart. We did not obtain any *Nfatc1-2A-GFPCre* mouse by NgAgo strategy. Scale bar, 100μm.

NgAgo Failed to Catalyze DNA-guided Genome Editing in 293T Cells and Zebrafish Embryos

Qing Wu[1,*], Lu Gao[1,*], Jing-Wei Xiong[1]

[1]Beijing Key Laboratory of Cardiometabolic Molecular Medicine and State Key Laboratory of Natural and Biomimetic Drugs, Institute of Molecular Medicine, Peking University, Beijing, 100871, China
(*QW and *LG contribute equally to this work)

Email address: jingwei_xiong@pku.edu.cn

Summary of findings:

Based on the methods by Gao et al., 2016 Nature Biotechnology, we were not able to detect any NgAgo-mediated genome editing in DYRK1A, EMX1 and HBA2 in cultured human 293T cells by using either NgAgo that was directly obtained from Han's lab or rat codon-optimized NgAgo (rNgAgo) we purchased (Figure 1). Similarly, we failed to detect any NgAgo-induced genome editing in gata4, gata5, gata6 and etv2 genes in zebrafish embryos by using either NgAgo that was directly obtained from Han's lab or zebrafish codon-optimized NgAgo (zNgAgo) we purchased (Figure 2). Therefore, our data suggest that NgAgo has no DNA-guided endonuclease activities in 293T cells and zebrafish embryos.

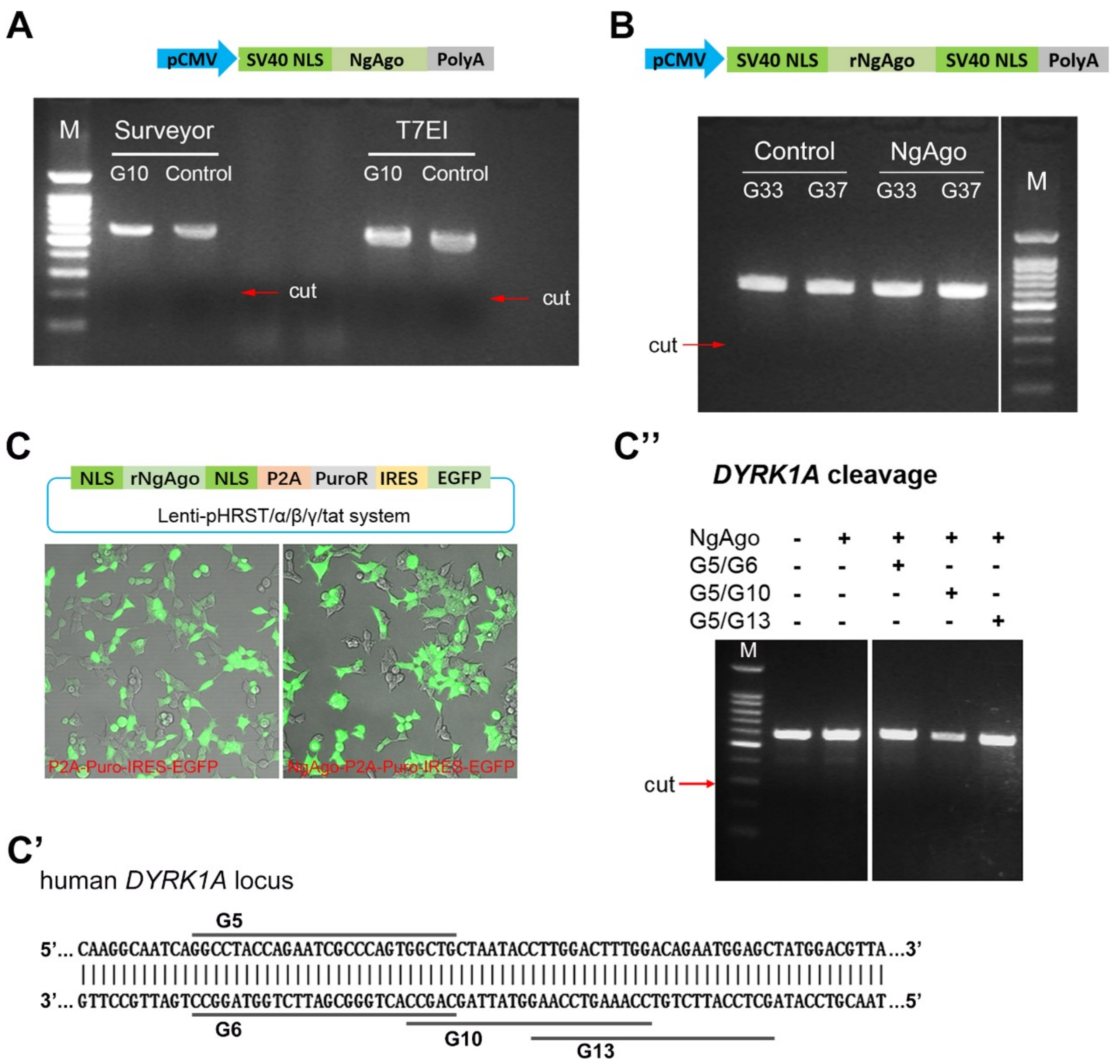

Figure 1. NgAgo failed to cleave DNA targets in culture human 293T cells.

(A) Schematic diagram of the NgAgo vector (top panel). Human 293T cells were transfected with NgAgo and G10 gDNA as reported by Gao et al., 2016 Nature Biotechnology. Two days later, the genomic DNA was isolated to investigate NgAgo-mediated gene editing by using Surveyor and T7E1 assays. G10 (gDNA sequence is identical to that by Gao et al., 2016), NgAgo/G10 gDNA for the human DYRK1A gene; the control (Control), normal 293T cell genomic DNA. Note no anticipated Surveyor or T7EI products (lower panel, red arrows), suggesting that NgAgo/G10 had no DNA-guided cleavage in DYRK1A. M, 100 bp DNA ladder.

(B) Schematic diagram of the rat codon-optimized NgAgo (rNgAgo) vector (top panel). Human 293T cells were transfected with rNgAgo /G33 gDNA or rNgAgo/G37 (G33 and G37 gDNA are identical to those by Gao et al., 2016) that are designed for human EMX1 and HBA2, respectively. The second transfection of gDNA was performed at 24 hours after initial transfection. After 24 hours, the genomic DNA was isolated to examine rNgAro-mediated cleavage by using T7E1 assay. Either rNgAgo/G33 or rNgAgo/G37, as well as controls only with G33 or G37 (Control) in 293T cells had no DNA-guided cleavages in their targeted genes (lower panel, red arrow), that is, no anticipated T7EI products. M, 100 bp DNA ladder.

(C, C' and C'') Lentiviral-mediated expression of rNgAgo has no DNA-guided cleavages in 293T cells. (C) Rat codon-optimized NgAgo (rNgAgo) was infected into 293T cells by using a lentiviral system (top panel). The efficiency of infection was examined by EGFP expression (green; lower panel). (C') Schematic showing guide DNA sequences (G5, G6, G10 and G13 are identical to those by Gao et al., 2016) for targeting human DYRK1A gene. (C'') Infected 293T cells with rNgAgo were transfected with a pair of gDNA (G5/G6, G5/G10, or G5/G13). After 24 hours, the second transfection of the same gDNA pair was performed. After another culture for 24 hours, the genomic DNA was isolated to examine rNgAgo-mediated genome editing by using T7E1 assay. rNgAgo with either G5/G6, G5/G10, or G5/G13 had no DNA guided cleavages, that is, no anticipated T7EI products (red arrow). M, 100 bp DNA ladder.

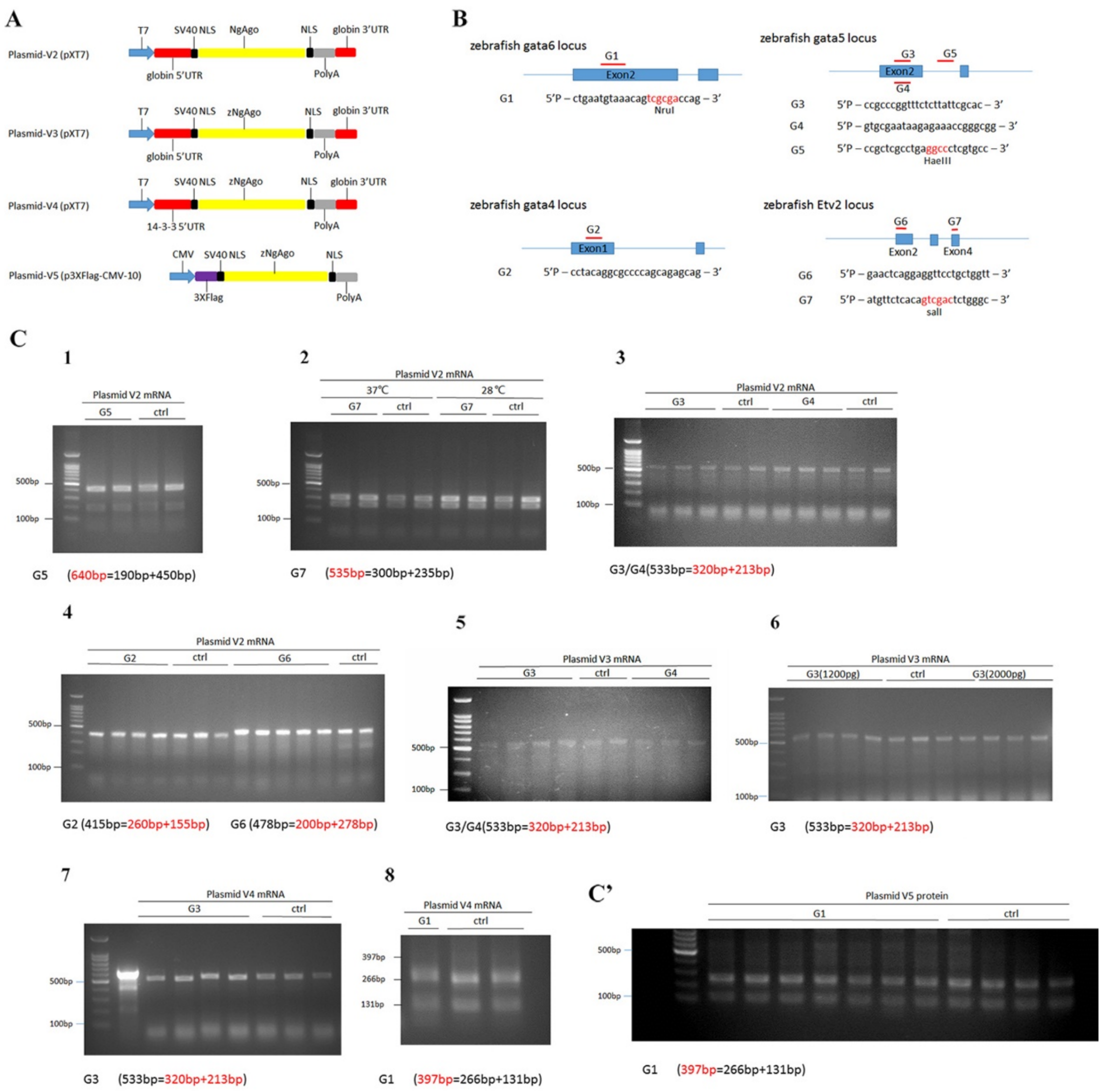

Figure 2. NgAgo has no DNA-guided endonuclease activities in zebrafish embryos. (A) Four types of the NgAgo vectors for *in vitro* synthesizing capped mRNA by using T7 polymerase, with different 5'-UTR or 3'-UTR (V2, V3, and V4), or for producing recombinant NgAgo proteins (V5). NgAgo, the same clone as reported by Gao et al., 2016 Nat Biotechnology; zNgAgo, zebrafish codon-optimized NgAgo. (B) Schematic diagram of the DNA guides for gatat4, gata5, gata6, and etv2 in zebrafish. The restriction enzyme sites in the targeted genes are used to facilitate genotyping and are marked in RED. For other targeted sites without restriction enzymes, we chose the T7E1 assays for genotyping. (C, C') Either restriction enzyme digestion or T7E1 assays on evaluating NgAgo-induced genome editing in targeted genes. "Red" PCR fragments indicate the expected bands if mutations were induced by NgAgo. (C) One-cell embryos were injected with about 600 pg mRNA from Plasmids V2-4, together with 200 pg gDNA per embryo (panels C1, C2, C3, C4, C5, C7, C8) or 1200 pg and 2000 pg G3 gDNA per embryo (panel C6); and with all culture temperature 28 ^{0}C, except with 37 ^{0}C or 28 ^{0}C (panel C2). (C') One-cell embryos were injected with 90 pg of zNgAgo proteins and 500 pg gDNA per embyo. zNgAgo proteins were expressed from plasmid V5 by OriGene Technologies. Note that either NgAgo or zNgAgo mRNA or proteins had any DNA-guided nuclease activities.
